# Extensive reorganization of the chloroplast genome of *Corydalis platycarpa*: A comparative analysis of their organization and evolution with other *Corydalis* plastomes

**DOI:** 10.3389/fpls.2022.1043740

**Published:** 2022-12-09

**Authors:** Gurusamy Raman, Gi-Heum Nam, SeonJoo Park

**Affiliations:** ^1^ Department of Life Sciences, Yeungnam University, Gyeongsan, Gyeongsan-buk, Republic of Korea; ^2^ Plants Resource Division, Biological Resources Research Department, National Institute of Biological Resources, Seo-gu, Incheon, Republic of Korea

**Keywords:** *Corydalis*, Plastome rearrangement, relocation, IR expansion, *accD*, *clpP*, *ndh*, divergent time

## Abstract

**Introduction:**

The chloroplast (cp) is an autonomous plant organelle with an individual genome that encodes essential cellular functions. The genome architecture and gene content of the cp is highly conserved in angiosperms. The plastome of *Corydalis* belongs to the Papaveraceae family, and the genome is comprised of unusual rearrangements and gene content. Thus far, no extensive comparative studies have been carried out to understand the evolution of *Corydalis* chloroplast genomes.

**Methods:**

Therefore, the *Corydalis platycarpa* cp genome was sequenced, and wide-scale comparative studies were conducted using publicly available twenty *Corydalis* plastomes.

**Results:**

Comparative analyses showed that an extensive genome rearrangement and IR expansion occurred, and these events evolved independently in the *Corydalis* species. By contrast, the plastomes of its closely related subfamily Papaveroideae and other Ranunculales taxa are highly conserved. On the other hand, the synapomorphy characteristics of both *accD* and the *ndh* gene loss events happened in the common ancestor of the *Corydalis* and sub-clade of the *Corydalis* lineage, respectively. The *Corydalis*-sub clade species (*ndh* lost) are distributed predominantly in the Qinghai-Tibetan plateau (QTP) region. The phylogenetic analysis and divergence time estimation were also employed for the *Corydalis* species.

**Discussion:**

The divergence time of the *ndh* gene in the *Corydalis* sub-clade species (44.31 – 15.71 mya) coincides very well with the uplift of the Qinghai-Tibet Plateau in Oligocene and Miocene periods, and maybe during this period, it has probably triggered the radiation of the *Corydalis* species.

**Conclusion:**

To the best of the authors’ knowledge, this is the first large-scale comparative study of *Corydalis* plastomes and their evolution. The present study may provide insights into the plastome architecture and the molecular evolution of *Corydalis* species.

## Introduction

In angiosperms, chloroplast (cp) genomes are highly conserved in terms of their structure, gene content, and gene arrangement contains a pair of inverted repeats (IRs) that separate with a large single-copy (LSC) and a small single-copy (SSC) region (Palmer, 1983; Palmer, 1985; Wicke et al., 2011; Maliga, 2014; Lee et al., 2016; Mower and Vickrey, 2018). The cp genome of angiosperms comprises roughly 80 protein-coding genes, which play a role in essential cellular functions and photosynthesis, along with 30 transfer and four ribosomal RNA genes (Bock, 2007). Among these, approximately seventeen genes were duplicated in the IR region. In addition, most of the land plant cp genome size varies from 110 to 170 kb, and the difference in cp size is frequently ascribed to extension, reduction, or loss of the IR region (Chumley et al., 2006; Wicke et al., 2011). The large-scale IR expansion is identified in the *Pelargonium transvallense* (Geraniaceae) (Weng et al., 2014), in which the IR enlarged higher than tripled (87.7 kb) compared to the typical size of the IR region (~25 kb). On the other hand, the IR region of two lineages of *Erodium* (Geraniaceae) (Blazier et al., 2016; Ruhlman et al., 2017), *Carnegiea gigantean* (Cactaceae) (Sanderson et al., 2015), *Tahina spectabilis* (Arecaceae) (Choi et al., 2019), the Putranjivoid clade of Malpighiales (Jin et al., 2020a), and IR-lacking clade (IRLC) of Papilionoideae (Fabaceae) (Palmer and Thompson, 1982) plastome size reduced significantly. Generally, gene arrangement is not often in most angiosperms plastomes (Frailey et al., 2018). If so, the plastome rearrangement is relatively small (Xu and Wang, 2021). Nevertheless, a large amount of rearrangement is rare, but it is infrequently present in a few lineages, namely Asteraceae (Jansen and Palmer, 1987; Kim et al., 2005; Sablok et al., 2019), Campanulaceae (Knox et al., 1993; Cosner et al., 2004; Knox, 2014; Knox and Li, 2017; Uribe-Convers et al., 2017), Fabaceae (Kolodner and Tewari, 1979; Palmer and Thompson, 1981; Lavin et al., 1990; Doyle et al., 1996; Cai et al., 2008; Martin et al., 2014; Schwarz et al., 2015; Wang et al., 2017), Geraniaceae (Palmer et al., 1987; Chumley et al., 2006; Guisinger et al., 2011; Weng et al., 2014; Roschenbleck et al., 2017; Weng et al., 2017), Oleaceae (Lee et al., 2007), Plantaginaceae (Zhu et al., 2016; Kwon et al., 2019; Asaf et al., 2020), and Poaceae (Palmer and Thompson, 1982; Doyle et al., 1992; Michelangeli et al., 2003; Burke et al., 2016; Liu et al., 2020).

In the family Papaveraceae, *Corydalis* belongs to the Fumarioideae subfamily. It comprises more than 465 species and is the largest genus in the Papaveraceae family (Zhang et al., 2008). Some *Corydalis* species have medicinal properties and have tremendous potential against hepatitis, tumor, muscular pain, and cardiovascular diseases (Luo et al., 1984; Zhang et al., 2016). The morphological characteristics of the *Corydalis* species are diversified and adapt to various habitats, such as grasslands, forests, riversides, shrubs, and cliffs. In addition, the *Corydalis* species can grow from sea level to more than 6,000 meters in elevation, which is of great interest to ecologists and evolutionary biologists (Niu et al., 2014; Niu et al., 2017). Moreover, these species are distributed widely from the north temperate regions, specifically Qinghai–Tibet Plateau (QTP), to southeast China, Myanmar, the Korean peninsula, and Japan. Xu and Wang (2021) reported that the *Corydalis* species had undergone severe and rapid differentiation (Xu and Wang, 2021). The *Corydalis* plastomes must have also experienced a sequence of genetic shifts to adapt to the radically altered environment. Therefore, ecology biologists are interested in understanding how the plastome structure and its content have fluctuated on a fine scale in the evolutionary period and when rare plastome rearrangements were derived, which is why these modifications occurred (Xu and Wang, 2021). Nevertheless, few *Corydalis* species have been reported since 2019, and a large-scale plastome rearrangement in their structure has been observed. Thus far, twenty *Corydalis* cp genomes have been sequenced, but most studies have reported only briefly. Among these, two research articles explained the genomes in detail. Xu and Wang (2021) used six, and Ren et al. (2021) used two *Corydalis* plastomes for comparative studies (Ren et al., 2021; Xu and Wang, 2021). On the other hand, there are no extensive comparative studies of *Corydalis* plastomes to understand their genome rearrangement patterns, such as inversion, relocation, expansion, and contraction of IR regions, and their molecular evolution patterns in detail. In addition, divergent time-related molecular studies of the *Corydalis* species could not be found. Therefore, a new plastome of *C. platycarpa* was sequenced, and detailed comparative genomic analyses of all the publicly available twenty *Corydalis* plastomes were conducted. Based on this, this study examined the complexity of the genome structure and rearrangement, gene content, gain/loss of genes and introns, repeats, RNA editing, nucleotide diversity, and adaptive evolution of the *Corydalis* plastomes. Furthermore, the phylogenetic position and divergent time of the *Corydalis* lineages were estimated.

## Materials and methods

### Genomic DNA isolation and *Corydalis* genome sequencing

Fresh leaves of *C. platycarpa* were sampled from Cheongok mountain, Bonghwa-gun, South Korea (geospatial coordinates: N37°4′9″, E128°57′47″). A voucher specimen (YNUH22C183) was deposited at Yeungnam University Plant Herbarium, Gyeongsan, South Korea. The total gDNA was extracted from the fresh *Corydalis* leaves by a modified CTAB method (Doyle, 1990). Next-generation sequencing was performed with an Illumina HiSeq2500 by Phyzen Ltd., South Korea. The paired-end (PE) library (2 × 150 bp) was constructed using TruSeq PCR free kit and then paired reads with 550 bp insert size were sequenced and ~3 GB of raw data were obtained. FastQC v0.11 (Andrews, 2010) was used to check the low-quality reads, which were removed using Trimmomatic 0.39 (Bolger et al., 2014).

### Assembly and annotation of the *Corydalis* chloroplast genome

For the *de novo* chloroplast (cp) genome assembly, the plastid-like reads were obtained from clean reads using the GetOrganelle pipeline v1.7.6.1 (Jin et al., 2020b). The filtered reads were then assembled using SPAdes v3.15.2 (Nurk et al., 2013) for the circular cp genome assembly in the paired-end mode. The assembled *C. platycarpa* cp genome coverage is 26,922×. The complete cp genome sequence and gene annotation were made using the online DOGMA program (Wyman et al., 2004) along with the cp genome annotations of *Nicotiana tabacum* (NCBI Reference sequence: NC_001879). Manual curation was carried out to adjust the start and stop codons of protein and ribosomal coding genes. The *Corydalis* cp genome circular map was drawn using OGDRAW v1.3.1 (Lohse et al., 2007). The annotated genome sequence was submitted to GenBank and assigned the accession number OP142703.

### Chloroplast genome sequence divergence and comparison

The newly sequenced *C. platycarpa* cp genome and other 20 publicly available *Corydalis* plastomes (**Supplementary Table S1**) were compared to determine the cp genome structure synteny and identify the possible rearrangements with the plastome of *N. tabacum* as a reference using Mauve v1.1.3 with the progressiveMauve algorithm (Katoh et al., 2019). A single IR region was used in this analysis. The schematic diagram was drawn manually based on their plastome structure to access the expansion/contraction of the LSC, IR, and SSC junctions of the 21 *Corydalis* plastomes. The entire plastome sequences of all the *Corydalis* were used to visualize the sequence similarity using mVISTA in Shuffle-LAGAN mode (Frazer et al., 2004), with the default parameters and *C. platycarpa* plastome used as a reference.

### Analyses of repetitive sequences

The simple sequence repeats (SSR) motifs were analyzed in the 21 plastomes of *Corydalis* using MISA v2.1 (Thiel et al., 2003) with the smallest number of repeats set to ten repetitions for mononucleotide SSRs, six repeat units for dinucleotide SSRs, and five repeat units for tri, tetra, penta, and hexanucleotide SSRs. The tandem repeats were searched using the Phobos Tandem Repeats Finder v1.0.6 with the parameters 1 for the match, −5 for mismatch and gap, and 0 for N positions (Mock et al., 2017). In addition, the forward, reverse, complement, and palindromic repeats were detected using REPuter with a Hamming distance of 3, 90% minimum sequence identity, and 30 bp of a minimal repeat size (Kurtz et al., 2001). For all these analyses, one copy of the IR region was used.

### Analyses of the genetic divergence

All 59 protein-coding genes were extracted and aligned individually using Geneious Prime (Biomatters, New Zealand) to evaluate the genetic divergence of the 21 *Corydalis* plastomes. All gaps and missing data were excluded before the analysis. The genetic divergence of 21 *Corydalis* plastomes was calculated by applying nucleotide diversity (π) and the total number of polymorphic sites in the DnaSP v6.12.03 (Librado and Rozas, 2009).

### Analysis of RNA editing sites in the protein-coding genes

The predictive RNA Editor for Plants (PREP) suite was applied to analyze the potential RNA editing sites in the protein-coding genes of the 21 *Corydalis* plastomes. The PREP-cp program has 35 reference genes explaining the RNA editing sites in the cp genomes (Mower, 2009). Therefore, 35 protein-coding genes of the *Corydalis* plastomes were utilized. In the present analysis, the cut-off value was set to 0.8.

### Analysis of substitution rate

The complete cp genome of *C. platycarpa* was compared with the other 20 *Corydalis* plastomes. The synonymous (K_S_) and non-synonymous (K_A_) substitution rates were analyzed by extracting the identical specific 59 functional protein-coding DNA sequences and translating them into protein sequences and aligning them independently using Geneious Prime (Biomatters, New Zealand). The synonymous and non-synonymous substitution rates were assessed in DnaSP v6.12.03 (Librado and Rozas, 2009). Similarly, the substitution analyses for all the 37 Ranunculales and all the Ranunculales (16 taxa) excluding the genus *Corydalis* cp genomes were compared.

### Analysis of positive selection

Positive selection analysis was performed based on substitution rate analyses of the 21 *Corydalis* plastomes. The site-specific model was employed using EasyCodeML v1.4 (Gao et al., 2019) to investigate the positive selection analysis. The 24 protein-coding gene sequence was aligned individually using the MAFFT alignment v1.5 (Katoh et al., 2019), and the maximum likelihood phylogenetic tree was built using RAxML v. 7.2.6 (Stamatakis et al., 2008). The codon substitution models, likelihood ratio test and the Bayes Empirical Bayes (BEB) analysis were conducted as described earlier.

### Analysis of phylogenetic tree

Thirty-seven cp genomes from the order Ranunculales were selected to construct a phylogenetic tree with *N. tabacum* selected as an outgroup, determine the location of the *C. platycarpa* in the order Ranunculales, and analyze the phylogenetic correlation of the *Corydalis* genus. The cp genome sequences of 29 species across the Papaveraceae family corresponding to two subfamilies (Fumarioideae and Papaveroideae) were downloaded. In addition, two chloroplast genomes of each subfamily of Berberidaceae, Ranunculaceae, Menispermaceae, and Ciraeasteraceae were included in this analysis (**Supplementary Table S1**). The 59 protein-coding genes shared by 38 plastomes were concatenated, aligned and saved in PHYLIP format using Clustal X v2.1 (Larkin et al., 2007). The Maximum-Likelihood (ML) tree was built using RAxML v7.2.6 with a General Time Reversible + Proportion Invariant model. One thousand non-parametric bootstrap replicates were performed to estimate the support of the data for each internal branch of the phylogeny (Stamatakis et al., 2008).

### Analysis of the evolutionary rate

Molecular divergence analysis of the Ranunculales lineages was performed with the Bayesian inference through Bayesian Markov chain Monte Carlo (MCMC) sampling implemented in BEAST v1.4 (Drummond and Rambaut, 2007) with a few modifications, as described earlier (Raman et al., 2021). A relaxed-clock log-normal model was applied using MCMC (500 million steps, sampled every 1000 generations, burn-in of 10%). A maximum clade credibility (MCC) tree was analyzed using TreeAnnotator v2.1.2 (Center for Computational Evolution, University of Auckland, New Zealand). Multiple calibration points were set for the divergence of the Berberidaceae subfamily, such as *Berberis bealei* at 88.94 mya (71.13–100.39 million years ago (mya), 78.22 mya (62.05–90.8 mya) for *Jeffersonia diphylla*, 62 mya (46.9–75.65 mya) for *Epimedium koreanum* and *Diphylleia cymosa*, 55.79 mya (38.65–73.14 mya) for *Nandina domestica* and 13.68 mya (8.05–21.75 mya) for *Caulophyllum robustum* and *Gymnospermium microrrhynchum*, which were employed with a log-normal distribution (Wang et al., 2016a).

## Results

### General features of the *Corydalis* chloroplast genome

**Figure 1:**
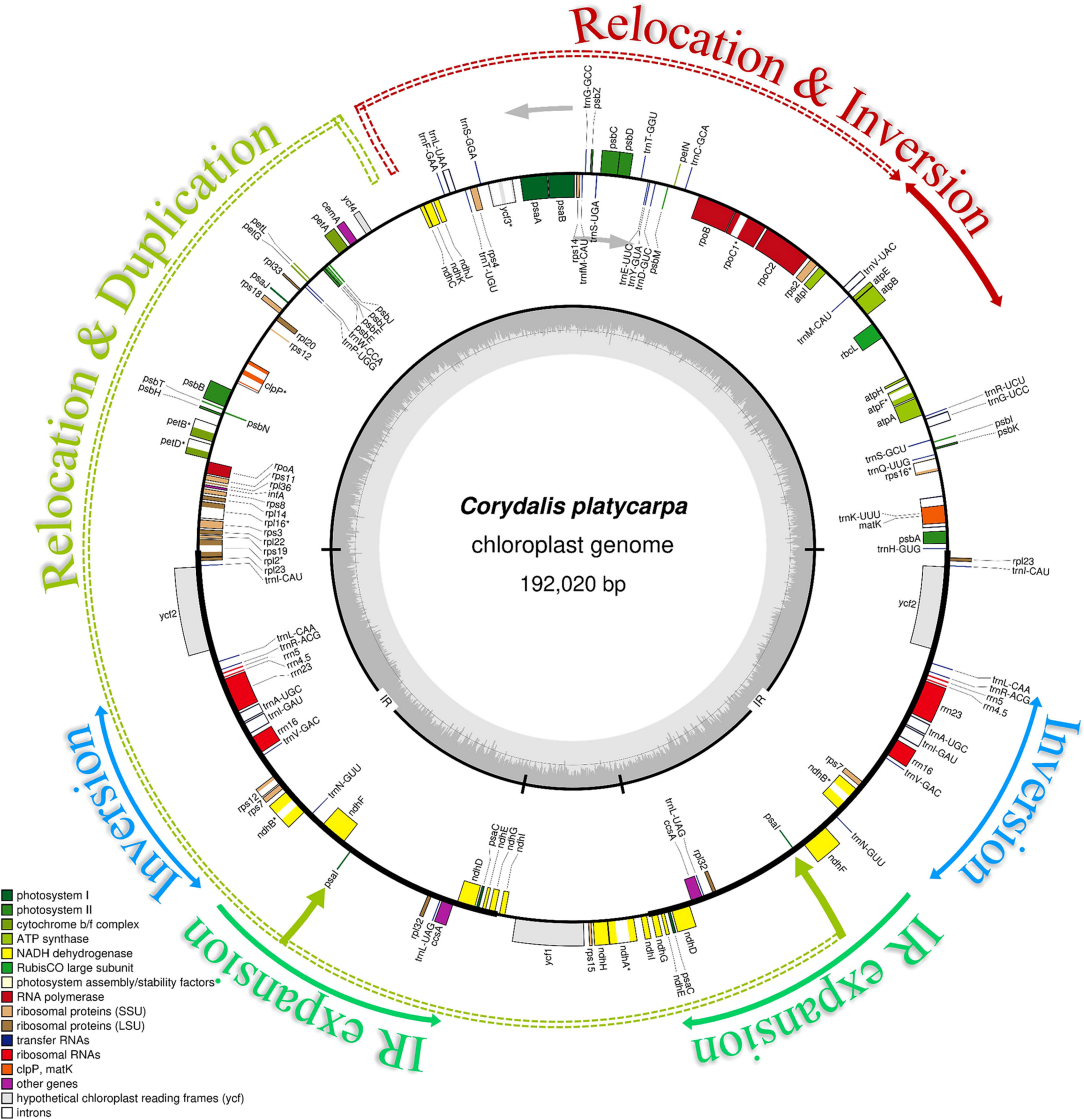
Circular chloroplast genome map of *Corydalis platycarpa*. Genes drawn outside the circle are transcribed clockwise, and those inside are counterclockwise. Genes belonging to different functional groups are color-coded. The darker grey in the inner circle shows the GC content, while the lighter grey shows the AT content.

**Figure 2:**
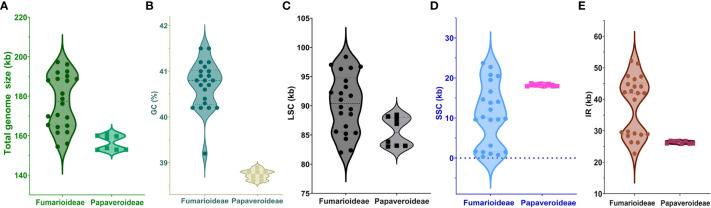
Visualization of **(A)** total genome size, **(B)** GC content, **(C)** LSC, **(D)** SSC, and **(E)** IR size of the Fumarioideae plastomes relative to Papaveroideae using a Violin plot.

The complete chloroplast (cp) genome sequence of *Corydalis platycarpa* (GenBank: OP142703) is 192,20 bp, with an inverted repeat (IR) of 42,640 bp separating a large single-copy (LSC) region of 96,492 bp and a small single-copy region of 10,247 bp (**Figure 1**). The average G+C content of the cp genome was 40.4%. The *C. platycarpa* cp genome includes 112 unique genes, such as 78 protein-coding, 30 tRNA, and four rRNA genes. In 112 genes, nine protein-coding and six tRNA genes contained a single intron, and *ycf3* and *rps12* encoded two introns, whereas *clpP* coded for three introns. Moreover, 26 genes were replicated in IR regions, fourteen involving protein-coding, eight tRNA, and four rRNA genes (**Supplementary Table S2**). The gene *accD* was entirely lost in the cp genome of *C. platycarpa*. In addition, the *C. platycarpa* was compared with other *Corydalis* species and other Fumarioideae (**Table 1**) and Papaveroideae plastomes (**Figure 2**; **Supplementary Table S3**). The average plastome size of the Fumarioideae was 177 kb, whereas the Papaveroideae was only 156.5 kb (**Figure 2A**). Similarly, the GC content of Fumarioideae and Papaveroideae was 40.7 and 38.7%, respectively (**Figure 2B**). In addition, the average length of the LSC region of Fumarioideae and Papaveroideae was 90.3 kb and 85.5 kb, and 10.78 and 18.2 kb of the SSC and 38 and 26.3 kb of the IR regions, respectively (**Figures 2C–E**).

**Table 1:** The basic genomic characteristics of 21 *Corydalis* plastomes.

S.	Species	Genome (bp)					Unique genes				Total genes		
No.	name	Total	LSC	IR	SSC	GC (%)	coding (cd)	tRNA (t)	rRNA (r)	Total	Gene Lost/Pseudogene	Duplicated (cd+t+r)	Total
**1**	***Corydalis platycarpa***	**192,020**	**96,492**	**42,640**	**10,247**	**40.4**	**78**	**30**	**04**	**112**	**01**	**26 (14 + 8+4)**	**138**
2	*Corydalis adunca*	196,128	92,145	47,226	9,531	41.0	71	30	04	105	08	22 (10 + 8+4)	127
3	*Corydalis conspersa*	187,810	92,280	47,375	780	40.8	67	30	04	101	12	25 (13 + 8+4)	126
4	*Corydalis davidii*	165,416	85,352	39,867	330	40.7	67	30	04	101	12	21 (9 + 8+4)	122
5	*Corydalis edulis*	154,395	81,999	26,250	19,504	40.2	77	29	04	110	03	18 (5 + 7+4)	128
6	*Corydalis fangshanensis*	192,554	98,393	42,263	9,635	40.3	78	30	04	112	01	26 (14 + 8+4)	138
7	*Corydalis filistipes*	169,237	97,016	28,741	14,640	41.2	77	30	04	111	02	17 (6 + 7+4)	128
8	*Corydalis hsiaowutaishanensis*	188,784	88,558	44,070	12,086	40.8	77	30	04	111	02	25 (13 + 8+4)	136
9	*Corydalis impatiens*	197,317	89,790	52,211	3,105	40.7	69	30	04	103	10	23 (11 + 8+4)	126
10	*Corydalis inopinata*	181,335	91,727	44,053	1,502	40.9	68	29	04	101	12	21 (9 + 8+4)	122
11	*Corydalis lupinoides*	178,650	85,220	46,377	1,506	40.8	67	29	04	100	13	19 (7 + 8+4)	119
12	*Corydalis maculata*	165,066	86,472	28,918	20,758	41.0	77	30	04	111	02	17 (5 + 8+4)	128
13	*Corydalis mucronifera*	176,217	85,579	44,778	1,082	40.2	72	30	04	106	07	26 (14 + 8+4)	132
14	*Corydalis namdoensis*	169,818	96,301	29,733	14,051	41.1	77	30	04	111	02	17 (6 + 7+4)	128
15	*Corydalis pauciovulata*	161,773	93,238	22,719	23,097	41.5	66	29	04	99	14	16 (5 + 7+4)	115
16	*Corydalis saxicola*	188,060	94,289	41,969	9,833	40.2	78	30	04	112	01	26 (14 + 8+4)	138
17	*Corydalis shensiana*	155,935	82,369	26,344	20,495	40.6	78	29	04	111	02	15 (4 + 7+4)	126
18	*Corydalis ternata*	170,483	88,722	29,514	22,733	41.2	75	30	04	109	04	17 (6 + 7+4)	126
19	*Corydalis tomentella*	190,198	96,701	41,955	9,636	40.3	77	29	04	110	03	26 (14 + 8+4)	136
20	*Corydalis trisecta*	164,354	91,046	28,345	16,618	41.5	75	29	04	108	05	17 (6 + 7+4)	125
21	*Corydalis turschanivoii*	161,534	89,414	29,143	13,834	40.9	77	30	04	111	02	17 (6 + 7+4)	128

Bold represents the species used in the present study.

### Comparative analysis of the *Corydalis* chloroplast genome structure

**Figure 3:**
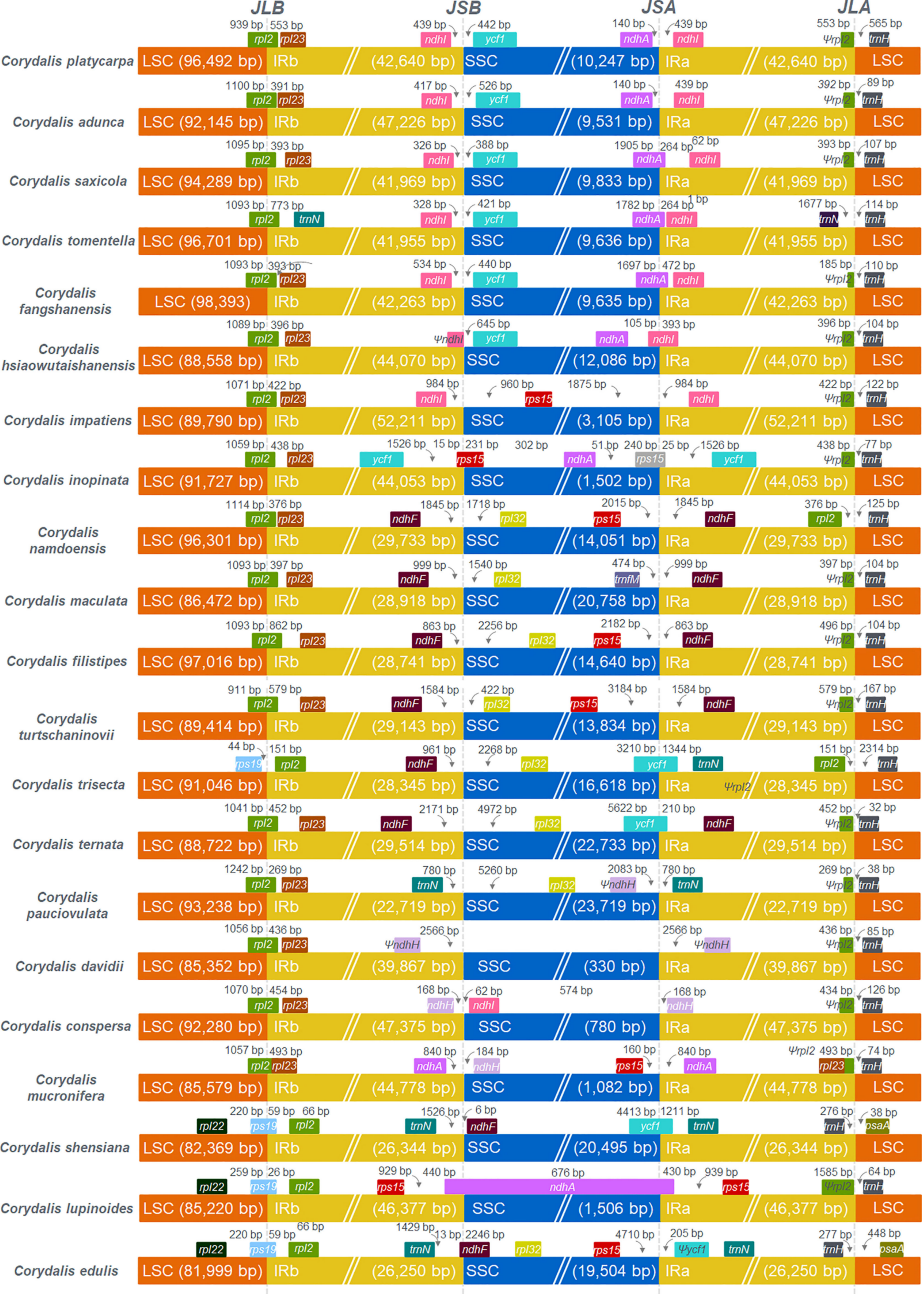
Comparison of the borders of LSC, SSC, and IR regions among 21 *Corydalis* chloroplast genomes. JLB indicates the junction line between LSC and IRb; JSB indicates the junction line between SSC and IRb; JSA indicates the junction line between SSC and IRa; JLA indicates the junction between LSC and IRa.

The mauve alignment revealed many rearrangements in the cp genome of the *C. platycarpa* and their relatives of *Corydalis* species (**Supplementary Figure S1**). Therefore, many events, namely, inversion, translocation, expansion, and contraction duplication, occurred in the SC and IR regions in the cp genomes of *Corydalis*. In the LSC region, the *rps16* gene was relocated within the LSC region of *C. adunca; rbcL* – *trnV*-UAC inversion and relocation occur in all the cp genomes of *Corydalis* except the species of *C. edulis* and *C. shensiana*. Similarly, the *ndhB* – *trnR*-ACG inversion was found in all the IR regions of the *Corydalis* cp genomes except the *C. edulis* and *C. shensiana* plastomes. In addition, a *ndhI* – *ycf1* inversion occurs in the *C. pauciovulata*. In addition, the expansion and contraction of the SC and IR boundaries of the 21 *Corydalis* cp genomes were evaluated using comparative analyses of the genes across the boundary regions (**Figure 3**). The *rps19* gene straddled the boundary of the LSC/IRB regions of the *C. shensiana*, *C. lupinoides*, and *C. edulis* cp genomes, whereas the *rpl2* gene straddled the LSC/IRB regions of the remaining 18 *Corydalis* cp genomes that lead to the length of LSC regions varies from 82 kb to 98.4 kb (**Figure 2**). In contrast, the IR regions are highly expanded in most *Corydalis* cp genomes ranging from 22.7 to 52.2 kb. The *ndhF* gene is spanned in the IRB/SSC region in the *C. shensiana* and *C. edulis* cp genomes. Nevertheless, *ndhI, ycf1, rps15, rpl32, trnN*, and *ndhH* genes traversed the remaining *Corydalis* cp genomes due to the relocation, inversion, and expansion of the IR regions. Correspondingly, contraction occurs in the SSC region in most of the *Corydalis* cp genomes (**Figures 2**, **3**) that affect the shuffling of the boundary genes (*ndhA, ndhI, rps15, trnfM, ycf1, trnN*, and *ndhA*) in the SSC/IRA regions. Similarly, most of the *Corydalis* genome encodes the *rpl2* pseudogene in the IRA/LSC boundary regions.

### Comparative analysis of the repeat sequences in the *Corydalis* cp genomes

**Figure 4:**
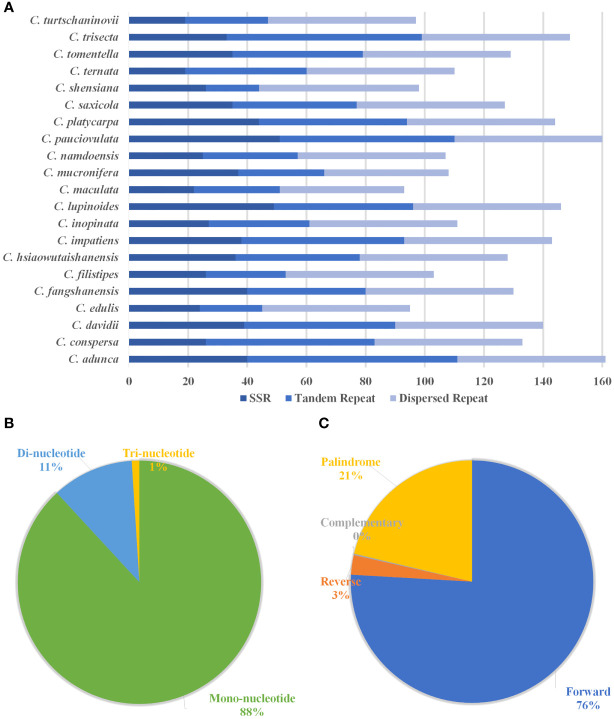
Histogram shows the number of repeats in 21 *Corydalis* chloroplast genomes. **(A)** The distribution of simple sequence repeats (SSRs), tandem repeats, and dispersed repeats in the 21 *Corydalis* plastomes. **(B)** Proportion of different SSR repeat types in the 21 plastomes of *Corydalis*. **(C)** The number of different types of dispersed repeats in the 21 *Corydalis* plastomes.

The results show that the total number of simple sequence repeats (SSRs) ranges from 19 (*C. ternata*) to 51 (*C. pauciovulata*), and the distribution of SSRs differs among the 21 plastomes of *Corydalis* (**Figure 4A**). Mononucleotides are the most frequent in the SSRs, distributing 88%, followed by dinucleotide and trinucleotides at 11% and 1%, respectively, in the *Corydalis* plastomes (**Figure 4B**). Among the mononucleotides, all the cp genomes occupy 96% of A and T type SSRs in their genomes, while most of the species lack dinucleotides, such as AG and CT trinucleotides, namely ATG, ATT, CAA, TTA, and TTG (**Supplementary Table S4**). Similarly, the distribution of tandem repeats in the *Corydalis* cp genomes ranges from 18 to 71. In addition to SSRs and tandem repeats, 1038 dispersed repeats are identified using REPuter (**Figure 4A**; **Supplementary Table S4**). Among the *Corydalis* cp genomes, the forward (76%), palindrome (21%) and reverse (3%) repeats are observed (**Figure 4C**).

### RNA editing site analysis in the *Corydalis* cp genomes

**Figure 5:**
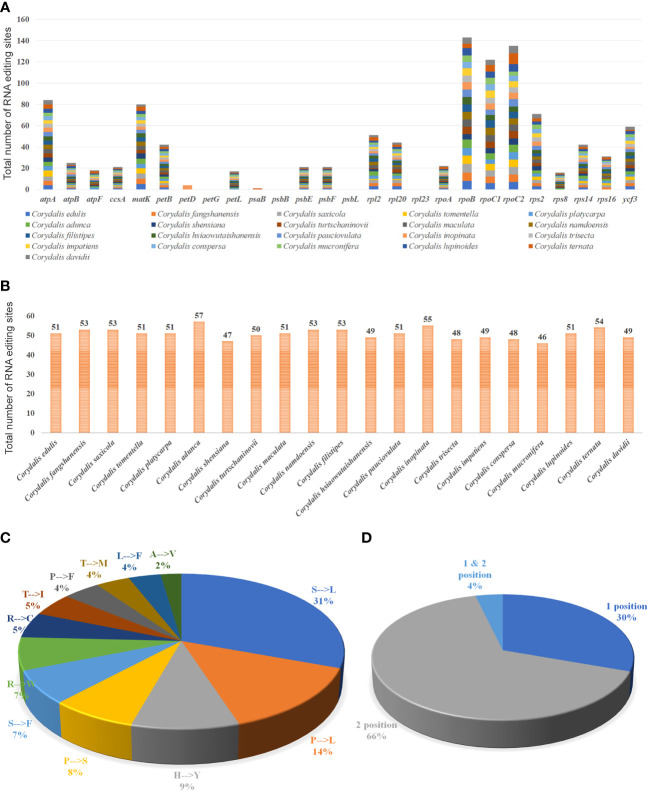
Analyses of RNA editing in the 35 protein-coding genes of the 21 *Corydalis* plastomes. **(A)** the distribution of RNA editing sites in the protein-coding genes of each *Corydalis* genome. **(B)** The number of RNA editing sites in each *Corydalis* cp genome. **(C)** Pie diagram represents the conversion percentage of amino acids in the RNA editing sites. **(D)** Represents the RNA editing site in the triplet codon of the nucleotide. S, serine; L, leucine; P, proline; H, histidine; Y, tyrosine; F, phenylalanine; R, arginine; W, tryptophan; C, cysteine; T, threonine; I, isoleucine; M, methionine; A, alanine; V, valine.

The possible RNA editing sites for 35 protein-coding genes were predicted by the PREP suite in the 21 *Corydalis* cp genomes. One thousand and seventy RNA editing sites were detected in their coding genes (**Figure 5A**; **Supplementary Table S5**). The number of editing sites varied from the 46 (*C. mucronifera*) to 57 (*C. adunca*) (**Figure 5B**; **Supplementary Table S5**). Among the 35 protein-coding genes of the *Corydalis* plastomes, the *rpoB* gene encoded the highest RNA editing sites (143), followed by *rpoC2* (135), *rpoC1* (122), *atpA* (84), *matK* (80), *rps2* (71), *ycf3* (59), *rpl2* (51), *rpl20* (44), and *petD* (42) (**Figure 5A**; **Supplementary Table S5**). In the RNA editing sites, 31% of sites converted serine to leucine, followed by 14% of proline to leucine, 9% of histidine to tyrosine, 8% of proline to serine, 7% of serine to phenylalanine, and 7% of arginine to tryptophane amino acids (**Figure 5C**; **Supplementary Table S5**). All predictable RNA editing sites are cytosine to uracil (C–U) transitions, the maximum of which are situated at the second codon position (66%), followed by the first codon position (30%), first and second codon position (4%), besides no transitions at the third codon position (**Figure 5D**; **Supplementary Table S5**).

### Sequence divergence analysis in the *Corydalis* cp genomes

**Figure 6:**
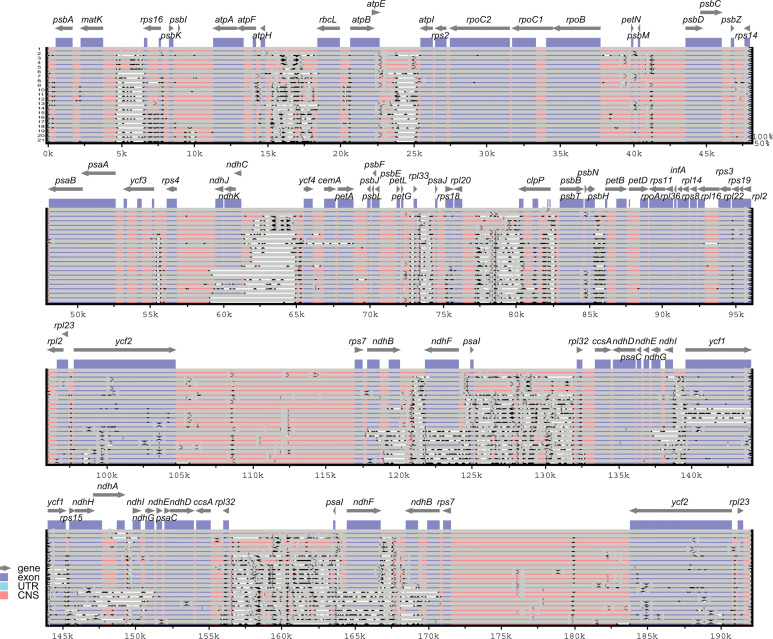
mVISTA-based sequence identity plot of 21 *Corydalis* plastomes with *C. platycarpa* as a reference. The gray arrows indicate the direction of the gene transcription. The y-axis represents the percent identity ranging from 50 to 100% is represented by the vertical scale. Coding and non-coding regions are colored purple and pink, respectively. 1. *Corydalis platycarpa*; 2. *Corydalis adunca*; 3. *Corydalis saxicola*; 4. *Corydalis tomentella*; 5. *Corydalis fangshanensis*; 6. *Corydalis hsiaowutaishanensis*; 7. *Corydalis impatiens*; 8. *Corydalis inopinata*; 9. *Corydalis namdoensis*; 10. *Corydalis maculata*; 11. *Corydalis filistipes*; 12. *Corydalis turtschaninovii*; 13. *Corydalis trisecta*; 14. *Corydalis ternata*; 15. *Corydalis pauciovulata*; 16. *Corydalis davidii*; 17. *Corydalis conspersa*; 18. *Corydalis mucronifera*; 19. *Corydalis shensiana*; 20. *Corydalis lupinoides*; 21. *Corydalis edulis*.

**Figure 7:**
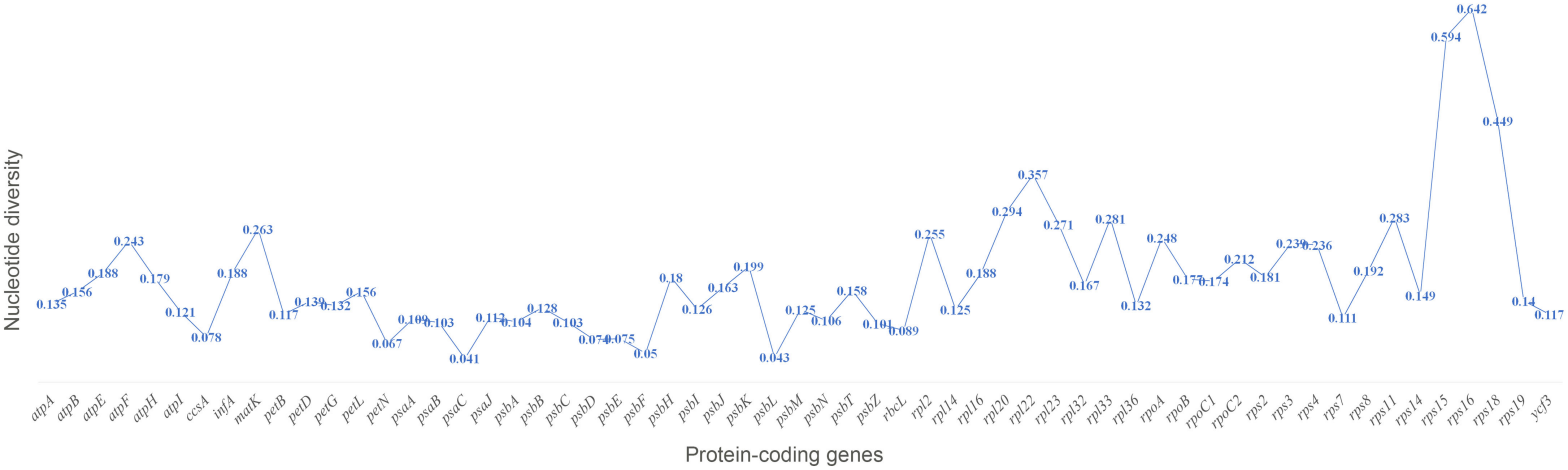
Percentage of variable characters (SNPs) in the protein-coding genes of 21 *Corydalis* plastomes.

The sequence divergence of all the 21 plastomes of *Corydalis* was analyzed using mVISTA and sequence identity plots constructed (**Figure 6**) with the annotated cp genome of *C. platycarpa* as the reference. The results showed that the ribosomal RNA genes in the IR regions were highly conserved and less divergent than the other coding and non-coding sequences in the LSC, SSC, and IR regions. In addition, the nucleotide diversity (Pi) of 59 protein genes in the *Corydalis* cp genomes was calculated. All 59 genes were highly variable regions (>0.03) that are associated with photosynthetic, transcription, and translational processes (**Figure 7**). Among these 59 genes, *psaC* has the lowest Pi value (0.041), and *rps16* has the highest Pi value 0.642.

### Adaptive evolution analysis in the *Corydalis* cp genomes

**Figure 8:**
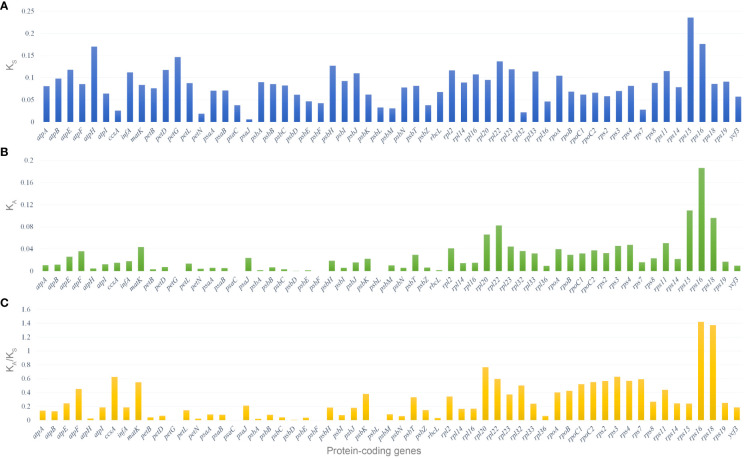
Selective pressure of 59 protein-coding genes in the 21 *Corydalis* plastomes. **(A)** K_S_, rate of synonymous substitution; **(B)** K_A_, rate of non-synonymous substitution; **(C)** K_A_/K_S_, rate of non-synonymous vs. synonymous substitution.

Fifty-nine shared protein-coding genes of all the 21 *Corydalis* plastomes were used for synonymous (K_S_) and non-synonymous (K_A_) substitution rates. The results showed that most protein-coding genes have relatively high average K_S_ values (>0.05) except the *ccsA*, *petN*, *psaJ*, *psbE*, *psbF*, *psbL*, *psbM*, *psbZ*, *rpl32*, *rpl36*, and *rps7* genes (**Figure 8A**; **Supplementary Table S6**). In the same way, most of the protein-coding genes are comparatively high average K_A_ values (>0.02) except *atpA*, *atpB*, *atpI*, *ccsA*, *infA*, *petB*, *petD*, *petG*, *petL*, *petN*, *psaA*, *psaB*, *psaC*, *psbA*, *psbB*, *psbC*, *psbD*, *psbE*, *psbF*, *psbH*, *psbI*, *psbJ*, *psbL*, *psbM*, *psbN*, *psbZ*, *rbcL*, *rpl14*, *rpl16*, *rpl36*, *rps7*, *rps19*, and *ycf3* genes (**Figure 8B**; **Supplementary Table S6**). The protein-coding genes, *rps16* and *rps18*, show the highest average K_A_/K_S_ ratio of 1.42 and 1.37, respectively. On the other hand, the K_A_/K_S_ ratios of all the protein-coding genes ranged from 0 to 1.42, with an average ratio of only 0.28 (**Figure 8A**; **Supplementary Table S6**). Similarly, all the 37 Ranunculales taxa were analyzed for substitution analysis and revealed that the K_A_/K_S_ ratios of all the protein-coding genes differed from 0 to 6.83, with an average ratio of 0.21 (**Supplementary Figure S2**; **Supplementary Table S7**). Furthermore, the substitution analysis of all the 59 protein-coding genes of Ranunculales, excluding the genus *Corydalis* (16 taxa), revealed that the K_A_/K_S_ rate varied from 0 to 0.89, with the average rate of 0.13 (**Supplementary Figure S3**; **Supplementary Table S8**).

Suppose the substitution ratio of the specific protein-coding genes among two cp genomes or the whole genomes is > 1.0. In that case, these genes are considered to be under positive selection. Therefore, the K_A_/K_S_ (ω) ratio of 24 protein-coding genes is > 1.0, and they were analyzed for selective pressure events. The ω_2_ ratio of 24 protein-coding genes ranges from 1.0 – 107.1382 in the M2a model (**Supplementary Table S9**). Bayes empirical Bayes (BEB) analysis was employed to assess the position of coherent selective sites in the 24 protein-coding genes utilizing the M7 vs. M8 model and determine that seventeen sites under possibly positive selection in the four protein-coding genes (*rpl20* – 2; *rpl22* – 3; *rpl23* – 1; *rps2* – 2; *rps3* – 1; *rps4* – 3; *rps11* – 1; *rps14* – 2 and *rps18* – 2) with posterior probabilities >0.95 and 21 sites (*ccsA* – 1; *psbJ* – 1; *psbK* – 1; *rpl20* – 1; *rpl22* – 3; *rps3* – 3; *rps4* – 5; *rps7* – 1; *rps8* – 1; *rps11* – 1 and *rps16* – 3) >0.99 (**Supplementary Table S9**). The positive selection models’ likelihood ratio test (LRT) statistics against their null models (2ΔLnL) for all 59 genes of 21 *Corydalis* species were evaluated. The 2ΔLnL value ranged from 1.450266 – 115.737378 (**Table 2**). In contrast, the protein-coding genes *atpB*, *atpE*, *atpF*, *matK*, *psbH*, *psbT*, *rpl16*, *rpl33*, and *rps15* did not positively the encode selected sites in their genes.

**Table 2:** Comparison of the likelihood ratio test (LRT) statistics of positive selection models against their null models (2ΔLnL) for across all *Corydalis* species.

Protein-coding genes	Comparison between models	2ΔLnL	*d.f.*	*p-*value
*atpB*	M0 vs M3	64.72463	4	0
	M1 vs M2a	0	2	1.0
	M7 vs M8	12.88498	2	0.001592437
	M8a vs M8	1.29917	1	0.254364888
*atpE*	M0 vs M3	8.40558	4	0.077801468
	M1 vs M2a	0	2	1.0
	M7 vs M8	1.450266	2	0.495279515
	M8a vs M8	0.009460	1	0.922517911
*atpF*	M0 vs M3	18.862202	4	0.000836479
	M1 vs M2a	1.628380	2	0.442998011
	M7 vs M8	2.077926	2	0.353821405
	M8a vs M8	1.641228	1	0.200149937
*ccsA*	M0 vs M3	33.063826	4	0.000001159
	M1 vs M2a	5.483712	2	0.064450615
	M7 vs M8	5.516278	2	0.063409664
	M8a vs M8	5.483310	1	0.019198871
*matK*	M0 vs M3	50.242330	4	0
	M1 vs M2a	3.937154	2	0.139655445
	M7 vs M8	5.304254	2	0.070501098
	M8a vs M8	4.425530	1	0.035405116
*psbH*	M0 vs M3	7.275126	4	0.122043958
	M1 vs M2a	3.999999	2	0.999998000
	M7 vs M8	0.103072	2	0.949769458
	M8a vs M8	0.547220	1	0.459455831
*psbJ*	M0 vs M3	61.838488	4	0
	M1 vs M2a	58.694068	2	0
	M7 vs M8	59.780422	2	0
	M8a vs M8	59.755664	1	0
*psbK*	M0 vs M3	64.659550	4	0
	M1 vs M2a	64.659888	2	0
	M7 vs M8	64.735386	2	0
	M8a vs M8	64.773896	1	0
*psbT*	M0 vs M3	1.969244	4	0.741415908
	M1 vs M2a	0	2	1.0
	M7 vs M8	0.001906	2	0.999047454
	M8a vs M8	0.030365	1	0.861662368
*rpl16*	M0 vs M3	12.517974	4	0.013887770
	M1 vs M2a	0	2	1.0
	M7 vs M8	2.433942	2	0.296125775
	M8a vs M8	0.191028	1	0.662062396
*rpl20*	M0 vs M3	82.314682	4	0
	M1 vs M2a	20.167634	2	0.000041750
	M7 vs M8	21.600572	2	0.000020394
	M8a vs M8	21.441600	1	0.000003648
*rpl22*	M0 vs M3	123.460832	4	0
	M1 vs M2a	26.045432	2	0.00002210
	M7 vs M8	27.377434	2	0.000001135
	M8a vs M8	26.165314	1	0.000000313
*rpl23*	M0 vs M3	28.822982	4	0.000008492
	M1 vs M2a	3.272348	2	0.194723632
	M7 vs M8	4.449512	2	0.108093790
	M8a vs M8	3.723888	1	0.053639334
*rpl33*	M0 vs M3	13.382412	4	0.009550819
	M1 vs M2a	0.117402	2	0.942988681
	M7 vs M8	1.578368	2	0.454215284
	M8a vs M8	0.133798	1	0.714526193
*rps2*	M0 vs M3	56.521166	4	0
	M1 vs M2a	13.538212	2	0.001148721
	M7 vs M8	14.574584	2	0.000684178
	M8a vs M8	13.592536	1	0.000227087
*rps3*	M0 vs M3	120.920040	4	0
	M1 vs M2a	35.870314	2	0.000000016
	M7 vs M8	36.737234	2	0.000000011
	M8a vs M8	35.237912	1	0.000000003
*rps4*	M0 vs M3	164.241576	4	0
	M1 vs M2a	37.675708	2	0.000000007
	M7 vs M8	39.165622	2	0.000000003
	M8a vs M8	38.475640	1	0.000000001
*rps7*	M0 vs M3	122.425414	4	0
	M1 vs M2a	112.395174	2	0
	M7 vs M8	115.737378	2	0
	M8a vs M8	115.361240	1	0
*rps8*	M0 vs M3	126.748522	4	0
	M1 vs M2a	75.558924	2	0
	M7 vs M8	76.534240	2	0
	M8a vs M8	71.439114	1	0
*rps11*	M0 vs M3	56.457308	4	0
	M1 vs M2a	9.114974	2	0.010488383
	M7 vs M8	9.201318	2	0.010045214
	M8a vs M8	9.043680	1	0.002636045
*rps14*	M0 vs M3	27.682154	4	0.000014466
	M1 vs M2a	3.7647420	2	0.152228743
	M7 vs M8	5.0489140	2	0.080101796
	M8a vs M8	3.764736	1	0.052344121
*rps15*	M0 vs M3	0	4	1.0
	M1 vs M2a	1.420000	2	0.999929003
	M7 vs M8	1.680000	2	0.999916004
	M8a vs M8	0.025534	1	0.873043652
*rps16*	M0 vs M3	29.979244	4	0.000004942
	M1 vs M2a	20.030108	2	0.000044722
	M7 vs M8	19.907798	2	0.000047542
	M8a vs M8	19.578680	1	0.000009654
*rps18*	M0 vs M3	28.119374	4	0.000011797
	M1 vs M2a	9.563052	2	0.008383196
	M7 vs M8	11.694356	2	0.002888038
	M8a vs M8	9.638832	1	0.001905063

### Phylogenetic analysis of the Ranunculales

**Figure 9:**
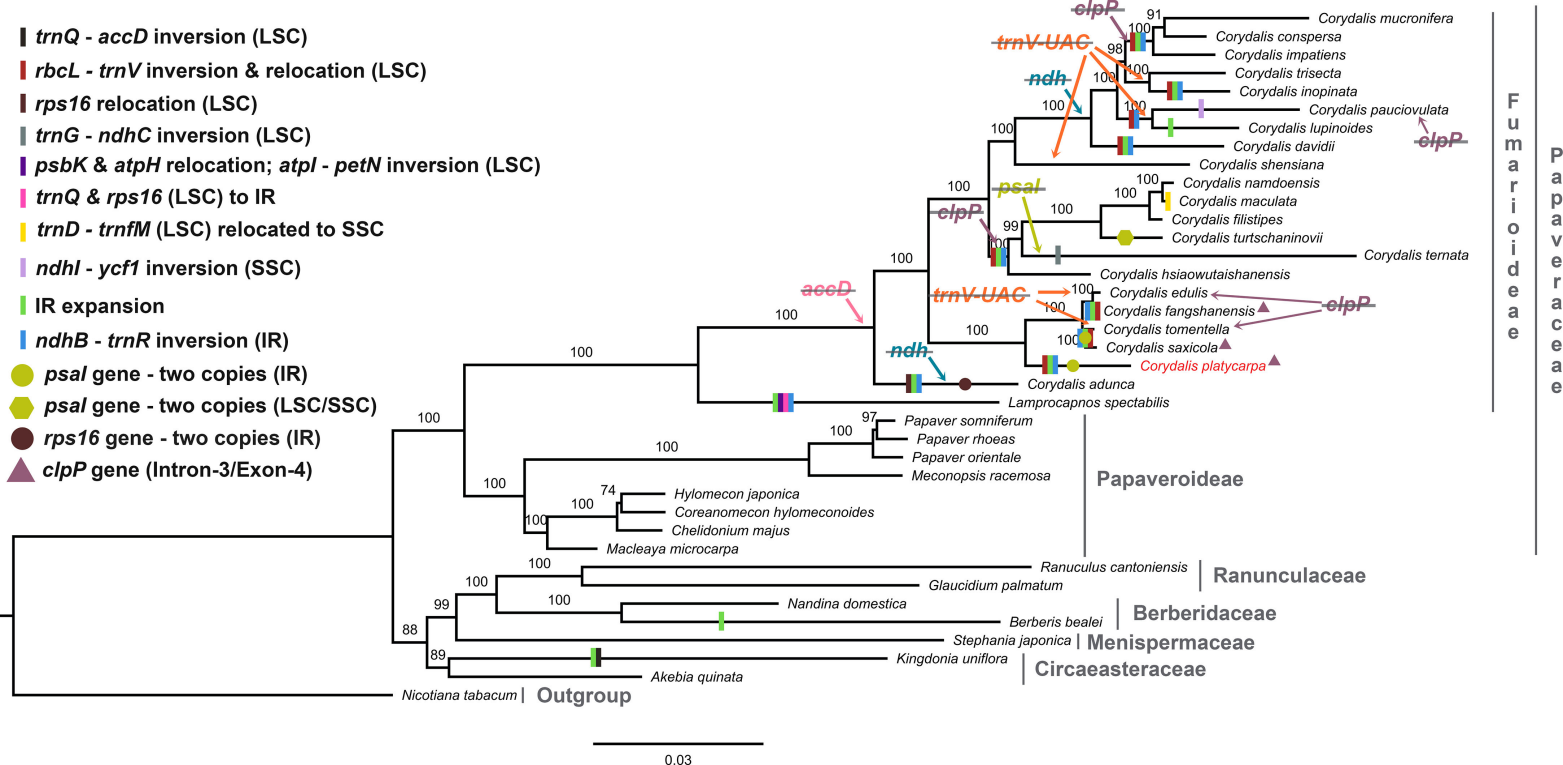
Maximum likelihood (ML) tree for 38 taxa based on 59 common plastid protein-coding genes. Values above the branches represent the maximum likelihood bootstrap value.

In the present study, 59 concatenated protein-coding genes were used to investigate the phylogenetic relationship of Ranunculales. All the Ranunculales species were clustered into two lineages (clade I and II). In the Papaveraceae lineage, it was grouped into Fumarioideae and Papaveroideae clades. All the *Corydalis* species were clustered into three clades, and *C. adunca* is the basal group in the tree (**Figure 9**). *C. platycarpa* is the sister to *C. Saxicola*, *C. tomentella*, *C. fangshanensis*, and *C. edulis* and formed one clade. *C. ternata*, *C. turtschaninovii*, *C. flistipes*, *C. maculata*, and *C. namdoensis* formed another clade, whereas *C. davidii*, *C. lupinoides*, *C. pauciovulata*, *C. inopinata*, *C. trisecta*, *C. impatiens*, *C. conspersa*, and *C. mucronifera* formed the third clade. All the *Corydalis* species were supported with strong bootstrap values.

### Molecular clock analysis of the Ranunculales

**Figure 10:**
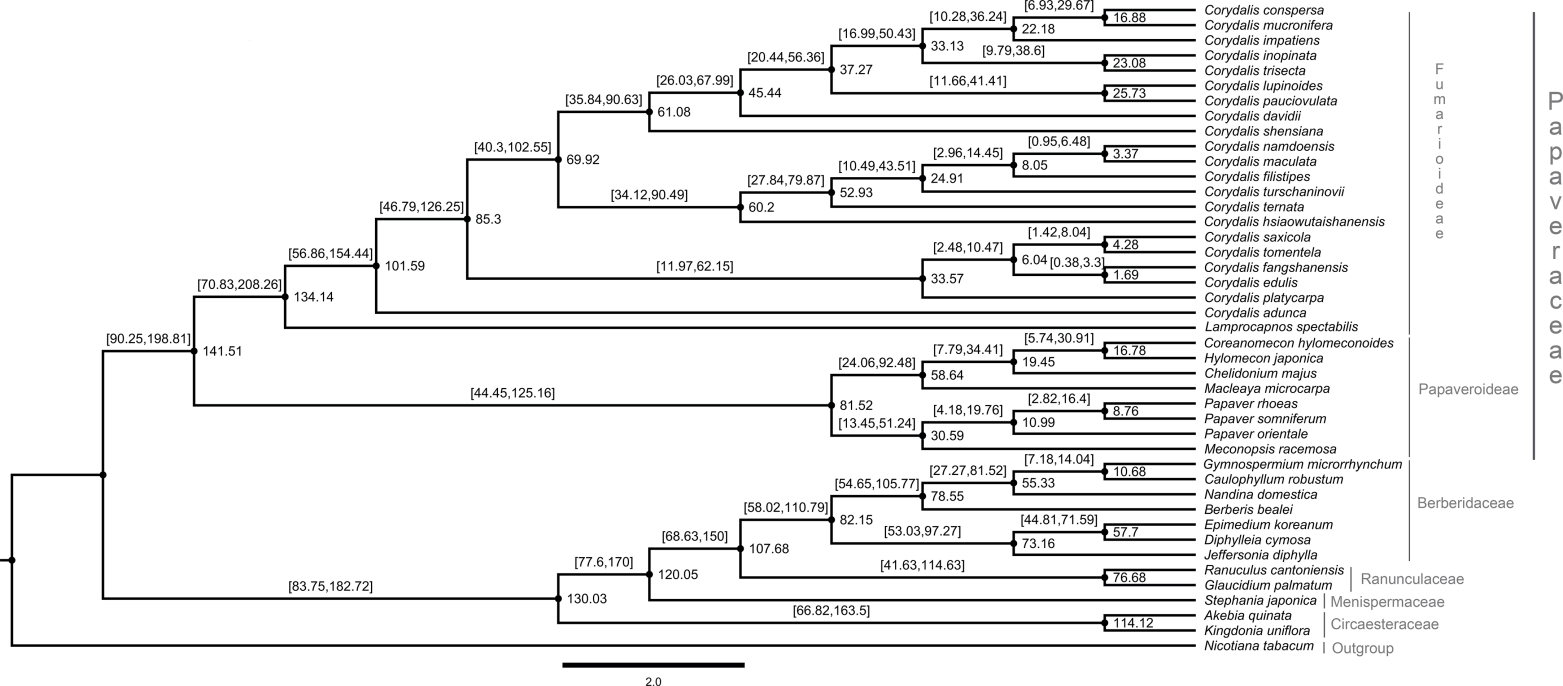
Estimation of the divergence time of *Corydalis* species using BEAST based on 59 plastid protein-coding genes of 42 Ranunculales and one outgroup species. The estimated mean ages are shown near the nodes, and blue bars represent 95% high posterior density.

The dataset for 59 protein-coding genes of 37 Ranunculales species was used to estimate the divergent time for the *Corydalis* species. Owing to a lack of calibration points, five other species of Ranunculales were also included. The divergent time was estimated using the previous data of the Ranunculales, which are similar to those obtained in the present study. Among order Ranunculales, the families Circaesteraceae, Menispermaceae, Ranunculaceae, and Berberidaceae diverged 138.13 million years ago (mya) (95% highest posterior density [HPD]: 198.81–90.25 mya). In the Papaveraceae family, Fumarioideae and Papaveroideae diverged 181.08 mya (95% HPD: 272.02–112.44 mya) and 155.65 mya (95% HPD: 223.22–98.61 mya), respectively (**Figure 10**). The chronogram resulting from a BEAST analysis showed that whole speciation events within *Corydalis* occurred from 98.6 to 1.51 mya. The *C. adunca* diverged from the ancestor of other remaining members of the *Corydalis* species at 98.6 mya (95% HPD: 154.44–56.86 mya). Among the *Corydalis*, the *C. platycarpa* diverged in the early Oligocene period (31.15 mya [95% HPD: 62.15–11.97].

## Discussion

*Corydalis* is the largest genus within the Papaveraceae family and contains more than 465 species (Zhang et al., 2008). Thus far, 20 chloroplast genomes have been sequenced and analyzed. No extensive or comparative studies of the *Corydalis* plastomes have been conducted thus far. Therefore, in the current study, the cp genome of *C. platycarpa* was sequenced and characterized, and comparative studies were carried out with twenty other species of the *Corydalis* genus. The results showed that the cp genomes of *Corydalis* displayed the total genome and size of the LSC, SSC, and IR regions (**Figure 2**; **Supplementary Table S3**). The gene order and its contents and the GC% content varied and were unusually greater or less than other Papaveraceae cp genomes. The total cp genome size of the *Corydalis* ranged from 154.4 kb (*C. edulis*) to 197.3 kb (*C. impatiens*), and *C. platycarpa* was the fourth largest cp genome (192 kb) in the *Corydalis* and the fifth largest among the Ranunculales cp genomes (**Figure 2**; **Supplementary Table S3**). The average size of the *Corydalis* cp genomes is 176.5 kb. In addition, the typical size of the cp genomes of Fumarioideae (*Corydalis* + *Lamprocapnos*) is 177 kb. In contrast, among the Papaveraceae family, the average genome size of the Papaveroideae is only 156.5 kb. Similarly, the average length of the LSC, SSC, and IR of the Fumarioideae was found to be 90.3, 10.78, and 38 kb, respectively. By contrast, the average lengths of the LSC, SSC and IR regions of Papaveroideae were 85.5 kb, 18.2 kb, and 26.3 kb, respectively. The variation in the Furmarioideae was attributed to the expansion of IR regions in their genomes, leading to a shift of the SC genes into the IR regions (**Figure 3**). In particular, the IR region was extended into the SSC region in most *Corydalis* cp genomes. *C. impatiens* encodes the largest IR region (52.2 kb), and *C. davidii* contains the smallest SSC region (330 bp). This variation in the size of Fumarioideae cp genomes also affects their GC content. The GC content of the *C. pauciovulata* and *C. trisecta* is the highest (41.5%) in the Ranunuculales plastomes. Commonly, a high GC content imparts more stability to the genome than the AT. In addition, the larger amount of GC base pairs in the genome might impact their adaptation to various adverse environments. On the other hand, the high percentage of AT affects the gene order and its content in the Fumarioideae cp genomes.

In Ranunculales, many relocations, inversions, and rearrangements occurred with all the Fumarioideae plastomes except for *C. edulis*, *C. shensiana*, and *C. trisecta*. Moreover, at least eight events have occurred in all three LSC, SSC, and IR regions (**Figure 9**; **Supplementary Figure S1**) and the following events occurred in the LSC region: (i) a ~ 10 kb of *rbcL* – *trnV*-UAC inverted and relocated into the upstream of *atpH* and downstream of the *atpI *gene in the LSC region (all the Fumarioideae genomes except *Lamprocapnos spectabilis, C. adunca*, *C. edulis*, *C. shensiana* and *C. trisecta*); (ii) the *rps16* gene (LSC) relocated into the IR region (*C. adunca*) and *trnQ *-UUG – *rps16* (LSC) into the IR (*L. spectabilis*); (iii) ~16 kb of *trnG*-GCC – *ndhC* inverted and relocated into the upstream of *psbZ* and downstream of *psbJ* in the LSC region (*C. ternata*); (iv) ~7 kb of *psbK* – *atpH* relocated and ~15 kb of *atpI *- *petN* inverted in the LSC region (*L. spectabilis*) (Park et al., 2018); (v) ~7.5 kb of *trnD*-GUC – *trnfM* relocated into the SSC region (*C. maculata*). The following occurred in the SSC region: (vi) ~10.5 kb of *ndhI* – *ycf1* inverted in the IR region (*C. pauciovulata*): (vii) ~14.4 kb of *ndhB* – *trnR*-ACG inverted (except *C. edulis*, *C. shensiana* and *C. trisecta* in the Fumarioideae); (viii) IR expanded (except *C. edulis*, *C. shensiana*, *C. pauciovulata* and *C. trisecta* in the Fumarioideae). Among these eight events in Fumarioideae, seven events occurred in at least one species of the *Corydalis* cp genome (**Figure 9**). Furthermore, the translocation and IR expansion analyses were extended in the Ranunculales cp genomes. In the *Berberis bealei* (Berberidaceae) (Ma et al., 2013), the ~13 kb of the LSC region (*rps19* – *psbB*) was transferred to the IR region (**Figure 9**). Similarly, ~6 kb of the SSC region moved to the IR region, and ~50 kb inversion (*trnQ*-UUG – *accD*) occurred within the LSC region of the *Kingdonia uniflora* (Circaeasteraceae) (Sun et al., 2020) (**Figure 9**). In contrast, Xu and Wang (2021) reported that the *ndhB* – *trnR*-ACG inversion event occurred in the IR region of the common ancestor of Fumarioideae plastomes (Xu and Wang, 2021). This is due to the limitation of the six plastomes used in their comparative studies. In the present study, 21 *Corydalis* species and one *L. spectabilis* (Park et al., 2018) cp genome (Fumarioideae) were used for comparative analyses, suggesting that the *ndhB* – *trnR*-ACG event did not occur in *C. edulis*, *C. shensiana*, and *C. trisecta* in the Fumarioideae (**Figure 9**). Therefore, single or all the genome rearrangement/relocation events did not take place in the common ancestor of either the *Corydalis* genera or Fumarioideae clade. Earlier studies reported that several cp genome rearrangements and relocation are feasibly lowest common in angiosperms. To support the present study, previous studies reported similar rearrangement events, such as inversion and relocation of *trnV*-UAC – *rbcL* event in the Oleaceae (Lee et al., 2007) and Campanulaceae (Knox, 2014; Knox and Li, 2017; Uribe-Convers et al., 2017) and *trnQ *-UUG – *rbcL* in the *Circaeaster agrestis* and *K. uniflora* of Ranunculaceae (Sun et al., 2017; Sun et al., 2020) occurred independently in their plastomes rather than there being a common ancestor.

The gene content in the *Corydalis* plastomes was compared. Usually, the cp genome comprises 79 protein-coding genes (excluding *ycf15* and *ycf68* genes), 30 transfer and four ribosomal RNA genes (Raman et al., 2019; Raman and Park, 2022). On the other hand, the genus *Corydalis* varied from 66 (*C. pauciovulata*) to 78 protein-coding genes (*C. platycarypa, C. fangshanensis, C. saxicola*, and *C. shensiana*) in their plastomes (**Table 1**). All the *Corydalis* species lost *accD*, and some of the *Corydalis* lost *clpP*, *ndh*, *rps16*, *psaI*, and *trnV*-UAC genes in their plastomes (**Figure 9**; **Supplementary Table S10**). The tRNA and rRNA contents were the same in almost all species except for the loss of the *trnV*-UAC gene in a few *Corydalis* cp genomes (*C. edulis, C. inopinata, C. lupinoides, C. pauciovulata, C. shensiana, C. tomentella*, and *C. trisecta*). In contrast, *trnV*-UAC was reported to be highly conserved in all monocot plants, whereas tRNA^Lys^, tRNA^Ala^, tRNA^Ile^, tRNA^Sec^, tRNA^Pyl^ and suppressor tRNA were absent in most of the monocot cp genomes (Mohanta and Bae, 2017; Mohanta et al., 2019; Mohanta et al., 2020b). By contrast, the absence of all the tRNA genes in the monocots was highly conserved in the *Corydalis* and other closely related cp genomes. Overall, losses of a minimum of one gene to a maximum of fourteen genes occurred in the *Corydalis* plastomes (**Table 1**; **Supplementary Table S10**). Among the protein-coding genes, the *accD* gene was lost in all *Corydalis* plastomes. This event happened in the common ancestor of the *Corydalis* lineages (**Figure 9**). The *accD* encodes one of four subunits of the acetyl-CoA carboxylase enzyme (ACC), which is necessary for fatty acid biosynthesis (Elborough et al., 1996; Sasaki and Nagano, 2004). Moreover, this enzyme is involved in the first process. Kode et al., 2005 suggested that the loss of the *accD* is detrimental to the plants, as observed in a study of tobacco (Kode et al., 2005). Earlier studies confirmed that the missing *accD* gene in the plastome is relocated in the nucleus of angiosperms species, such as *Trifolium repens* (Magee et al., 2010), Campanulaceae (Rousseau-Gueutin et al., 2013), and *Platycodon grandiflorum* (Hong et al., 2017). Because there were no transcriptome data, this study could not confirm whether the cp-encoded *accD* gene was lost wholly or functionally, which was relocated to the nucleus in the genus *Corydalis*. Therefore, further transcriptome studies will be needed to confirm whether the plastid copy is relocated in the *Corydalis* nuclear genome.

Typically, the plastid DNA of most of the higher plants encodes eleven *ndh* genes (Maier et al., 1995; Yukawa et al., 2005) that produce *ndh* polypeptides, forming a thylakoid *ndh* complex (Sazanov et al., 1998; Casano et al., 2000). This *ndh* complex is similar to the mitochondrial complex I, which catalyzes the transfer of electrons from NADH to plastoquinone (Martin and Sabater, 2010). Among the eleven *ndh* genes in the plastomes, the *ndhC*, *ndhK*, and *ndhJ* genes are situated in one transcriptional unit (*ndhC-J* operon) in the LSC region of the plastome (Serrot et al., 2008). The genes *ndhH, ndhA, ndhI, ndhG, ndhE*, and *ndhD* are located in the SSC region (*ndhH-D* operon), which also includes the gene *psaC* (encodes a polypeptide of the photosystem I complex, PSI) between the genes *ndhE* and *ndhD* (Del Campo et al., 2000). In addition, the *ndhF* gene is represented in the SSC region, and two identical copies of the *ndhB* gene exist in IR regions (one on each). The *ndhF* gene and the two *ndhB* genes are possibly transcribed autonomously as monocistronic mRNAs (Martin and Sabater, 2010). In the present study, some *Corydalis* lineages that displayed a wide-ranging pseudogenization or absence of the *ndh* genes in their plastomes were identified (**Figure 9**; **Supplementary Table S10**). A similar plastome rearrangement event accompanied by either pseudogenization or a loss of *ndh* genes was also identified in other Ranunculales species, *K. uniflora* (Sun et al., 2017) and Orchidaceae species (Lin et al., 2015). These two events occured independently in these species. In addition, comparative analyses of 2511 cp genomes showed that anyone of the *ndh* gene losses occurred commonly in at least one species of all lineages, such as algae, bryophytes, eudicots, gymnosperms, magnoliids, monocots, protists and pteridophytes (Mohanta et al., 2020a). However, in the *Corydalis* plastomes, at least three to all (eleven genes) *ndh* genes were either pseudogenized or lost in the plastomes of *C. adunca, C. conspersa, C. davidii, C. impatiens, C. inopinata, C. lupinoides, C. mucronifera, C. pauciovulata*, and *C. trisecta plastomes* (**Figure 9**; **Supplementary Table S10**). Among the loss of *ndh* genes in the nine plastomes, the *ndhC* and *ndhF* loss occurred in the plastomes of all nine species (**Figure 9**; **Supplementary Table S10**). The *C. adunca* is basal for the remaining *Corydalis* species, and the *ndh* gene loss occurred in their genome. The remaining eight species formed a single clade in the phylogenetic tree, and the *ndh* gene loss occurred in this clade, suggesting that after divergence from the *C. shensiana*, it probably occurred in the common ancestor of this clade plastomes (**Figure 9**). This event appears to be a synapomorphy that occurred at the subgenus level in the *Corydalis* clade. It is probably associated with the rearrangement and relocation of SC and IR genes, boundary shift, and expansion of the IR regions in the *Corydalis* plastomes. Interestingly, all nine species (except *C. pauciovulata*) were distributed predominantly on the Qinghai–Tibet Plateau (QTP) regions. The photosynthetic systems (*ndh* genes) in these plants, might have been lost due to the high altitude conditions, such as low temperatures, strong winds, and low atmospheric pressure, and adapted to their ecological environment.

The *clpP* gene is a proteolytic subunit of the ATP-dependent Clp protease found in higher plant chloroplasts (Shikanai et al., 2001). Usually, the *clpP* encoded three exons spliced by two type II introns in the cp genome (Raman et al., 2019; Raman and Park, 2022). Earlier studies stated that the loss of introns of the *clpP* gene had been determined in the *Geranium*, legume, *Silene*, and *Hypericum* cp genomes (Erixon and Oxelman, 2008; Dugas et al., 2015; Park et al., 2017; Claude et al., 2022). In this study, the *clpP* gene loss also took place in some of the *Corydalis* species, such as *C. conspersa, C. mucronifera, C. impatiens, C. namdoensis, C. maculata, C. filistipes, C.turtschaninovii, C. ternata, C. hsiaowutaishanensis, C. edulis* and *C. tomentalla* (**Supplementary Table S10**). Among these, *C. conspersa, C. mucnifera*, and *C. impatiens* formed one clade in the phylogenetic tree using 59 protein-coding concatenated datasets and *C. namdoensis, C. maculata, C. filistipes, C.turtschaninovii, C. ternata*, and *C. hsiaowutaishanensis* formed another (**Figure 9**). Except for the *C. edulis* and *C. tomentella*, the *clpP* gene loss may have occurred in the common ancestor of these two clades at the subgenera level (**Figure 9**). On the other hand, it is essential to use additional species to understand the *clpP* loss in the genomes of *Corydalis*. This is supported by a similar type of *clpP* loss that occurred in the common ancestor of the Actinidiaceae family (*Clematoclethra, Actinidia*, and *Saurauia*) (Wang et al., 2016b). In contrast, a few *Corydalis* plastomes (*C. platycarpa, C. saxicola*, and *C. fangshanensis*) encoded four exons and three introns in the *clpP* gene (**Supplementary Figure S3**). In the *clpP*, there was a ~115 bp insertion after exon 1 in the gene leading to the formation of an additional intron in their plastomes. The inserted nucleotide sequence similarity between the three species was 73.5%. In addition, this insertion sequence in the *clpP* gene was analyzed using BLASTN, but reliable results could not be obtained. The acquisition of one extra intron in the *clpP* gene may be due to the selective pressure in their genome. This could play a role in the evolutionary maintenance of the group II introns and provide more stability to the genome (Petersen et al., 2011). Selective pressures may have been significant and undervalued in the evolution of spliceosomal introns from group II intron progenitors (Chalamcharla et al., 2010). To the best of the authors*’* knowledge, this rare event has not been identified in any other plastomes. Therefore, further studies will be needed to understand the molecular mechanisms of the *clpP* gene in their plastomes.

In addition, gene duplication also occurred in the *Corydalis* plastomes because of genome rearrangements and boundary shifts. The two copies of the *rps16* gene in the IR regions of *C. adunca* are replaced with *rrn16* in the LSC region, suggesting that at least two rearrangements might have occurred in their plastome simultaneously or independently (Xu and Wang, 2021). At the same time, the *rps16* gene is a pseudogene in the *C. ternata*. Similarly, two copies of the *psaI* gene were found in the IR regions of *C. platycarpa, C. saxicola*, and *C. tomentella*: one copy from LSC and another from SSC in the *C. turtschaninovii* cp genomes (**Figure 9**; **Supplementary Figure S4**). Typically the *psaI* gene is located upstream of *accD* and downstream of the *ycf4* gene in the plastomes. The *psaI* gene duplication might have occurred in their cp genomes and was inserted into the IR region. The *psaI* was copied into another IR region due to the copy correction mechanism. In addition, the pseudogenization of the *psaI* gene in the LSC region was identified. A double-strand break might have occurred between the *ndhK* and *psaI* region (which contains *ndhK*, *trnV*-UAC, *trnM*-CAU, *atpE, atpB, rbcL, accD*, and *psaI*). This leads to the excision and inversion of this fragment inserted between the LSC regions in *atpH* and *atpI*. During this process, *accD* gene loss may have occurred in these three species, but this hypothesis could not be concluded for the remaining *Corydalis* plastomes, which might have played a role in the transposon activity. On the other hand, there is no direct evidence of transposable elements with the *Corydalis* cp genome, even though they may have been present transiently.

The SSRs a significant role during genome rearrangements and the recombination process (Ogihara et al., 1988; Milligan et al., 1989; Cole et al., 2018). Therefore, this study analyzed the presence of SSRs in the *Corydalis* plastomes. The distribution of the SSRs in the plastomes of *Corydalis* was quite different from 19 to 51. In addition, the *Corydalis* plastomes distributed many repeats in their genome ranging from 93 to 161 (**Figure 4**; **Supplementary Table S4**). Moreover, the presence of repeat sequences does not correlate with their genome rearrangements and relocation events in *Corydalis*. The *C. edulis* has 95 repeat sequences and does not encode major rearrangement events in its genome (**Figures 4**, **6**, **9**; **Supplementary Table S4**). On the other hand, the significant events (inversion, relocation, gene loss, and IR expansion) occurred in the *C. maculata, C. turtschaninovii,* and *C. shensiana* plastomes that encoded similar numbers of repeats (~95) regions in their genomes (**Figures 4**, **9**; **Supplementary Table S4**). Generally, the RNA editing process arises in the mitochondrial genomes but is less common in the plastomes (Chen et al., 2011; Raman and Park, 2015; Raman et al., 2016). In addition, the seed plant has ~30–40 RNA editing sites in its plastomes (Stern et al., 2010). Nevertheless, all the *Corydalis* have similar numbers (~51) of RNA editing sites in their genomes (**Figures 5A–D**). This process mainly occurred in the second position, followed by the first position of the triplet codon (**Figure 5D**). In addition, ~45% of the amino acids were converted to leucine (**Figure 5C**). Previous studies also reported that C to U RNA editing in the second codon position occurred mainly in plant organelles to enhance the hydrophobic amino acid leucine frequency. Chen et al. (2011) also reported that the closely associated taxa usually contribute to more RNA editing sites due to the evolutionary process but not in this study.

The mVISTA and nucleotide diversity analysis results showed a high degree of variation in both coding and non-coding regions in the *Corydalis* plastomes (**Figures 6**, **7**). The K_A_/*K_S_* rate is associated with gene adaptive evolution, such as the positive and purification selection effects (Raman et al., 2020; Raman and Park, 2020). The genes under positive selection might result from natural selection and adaptation to the living environment (Raven et al., 2013; Raman et al., 2020; Raman and Park, 2020; Scobeyeva et al., 2021). Therefore, the substitution rates of all the independent protein-coding genes of 21 *Corydalis* species are averaged. The results showed that the photosynthetic, transcription and transcription-related genes show accelerated non-synonymous rates (**Figures 8A, B**). Furthermore, the ratio of K_A_/K_S_ (ω) showed that the majority of the protein-coding genes were less than 1, excluding *rps16* and *rps18* genes (**Figure 8C**). A separate analysis of synonymous and non-synonymous substitution rates was also conducted for all protein-coding genes. Similarly, the substitution analysis of 59 protein-coding genes of all Ranunculales taxa (37 taxa) showed that the K_A_/K_S_ ratio varies from 6.83, with an average ratio of 0.21 (**Supplementary Figure S2**; **Supplementary Table S7**). In contrast, the substitution analysis of all the Ranunculales except *Corydalis* taxa (16 taxa) revealed that the K_A_/K_S_ ratio of all these protein-coding genes varies from 0 to 0.89, with an average ratio of 0.13 (**Supplementary Figure S3**; **Supplementary Table S8**). This result indicates that all the Ranunculales cp genomes, excluding *Corydalis* taxa, are highly conserved. Therefore, if the ω value is more than 1.0 of the particular protein-coding genes between two plastomes, or the whole genomes of *Corydalis* taxa, these genes are considered to be under positive selection. Therefore, in the present study, 24 protein-coding genes were identified in the *Corydalis* plastomes under positive selection pressure events (**Table 2**; **Supplementary Table S9**). In the selective pressure events, six forms of photosynthesis, transcription and translation-related genes were characterized: (i) subunits of ATP synthase (*atpB, atpE*, and *atpF*); (ii) C-type cytochrome synthesis gene (*ccsA*); (iii) maturase (*matK*); (iv) subunits of photosystem II (*psbH, psbJ, psbK*, and *psbT*); (v) large subunits of the ribosome (*rpl16, rpl20, rpl22, rpl23*, and *rpl33*); (vi) small subunit of the ribosome (*rps2, rps3, rps4, rps7, rps8, rps11, rps14, rps15, rps16*, and *rps18*). Among these, fourteen genes (*ccsA, psbJ, psbK, rpl20, rpl22, rpl23, rps2, rps3, rps4, rps8, rps11, rps14, rps16*, and *rps18*) have positively selected sites, providing evidence of the adaptive evolution of proteins (**Supplementary Table S9**). Genes with various functions, such as genetic and photosynthetic systems, might play a crucial role in the adaptation to the terrestrial ecological environment (Xu et al., 2015; Xu et al., 2020) because most of the *Corydalis* species live at QTP high altitudes and various North, Central, and East Asia terrestrial regions (**Supplementary Table S11**) and must adapt to high rates of UV radiation, oxygen depletion conditions, temperature fluctuations, and drought stress conditions. Such genes can be a significant genetic foundation for evolutionary adaptation at the chloroplast level (Xu et al., 2020).

The cp genomes are significant genomic resources for reconstructing precise and high-resolution phylogenetic relationships and taxonomic positions in angiosperms (Jansen et al., 2005). In addition to the whole cp genomes, protein-coding genes have been used widely to determine the phylogenetic relationships at every taxonomic level (Li et al., 2017). The phylogenomic analysis showed two distinct clades, such as Papaveraceae and the rest of the Ranunuculales. These results are consistent with the previous results. All the *Corydalis* lineages are highly supported with a *>*97% bootstrap value in the phylogenetic tree, and *C. adunca* is an early divergence species for the remaining *Corydalis* species (**Figure 9**). No molecular age studies for *Corydalis* species have been reported. Therefore, the divergent times for the genus *Corydalis* were analyzed. The *Corydalis* is estimated to have originated at 98.6 mya (95% HPD: 154.44–56.86 mya) in the early upper Cretaceous period and diverged. It took approximately 16 mya to form the rest of the *Corydalis* species (**Figure 10**). *C. platycarpa, C. edulis, C. fangshanensis, C. saxicola, C. hsiaowutaishanensis, C. ternata , C. turschaninovii, C. filistipes, C. maculata, C. namdoensis*, and *C. shensiana*, distributed in east Asia evolved from 82.86 to 1.51 mya. The remaining eight species are *C. davidii, C. pauciovulata, C. lupoinoides, C. trisecta, C. inopinata, C. impatiens*, and *C. mucronifera* and *C. conspersa*, mainly distributed in the QTP regions. The uplift of the QTP from the period of 25 to 17 mya (Li and Fang, 1999; Wang et al., 2012) changed the environment of East Asia dramatically. The molecular age results of all the eight QTP region *Corydalis* species (44.31 mya [95% HPD: 67.99–26.03 mya] – 15.71 mya [95% HPD: 29.67–6.93 mya]) correlated very well with the uplift of the Qinghai–Tibet Plateau period. This may have caused the radiation of *Corydalis* species during this period. Nevertheless, more taxa will be needed to understand the genome architecture, evolution, and divergence of the *Corydalis* species.

## Conclusion

The complete chloroplast genome sequence of *Corydalis platycarpa* species was determined using a *de novo* assembly approach. This is the first comprehensive systematic analysis comparing the plastome rearrangement features and adaptive evolution and inferring phylogenetic and molecular clock relationships using the plastome data of *Corydalis* and its relatives in detail. The comparative analysis showed that Fumarioideae species exhibited high rearrangements, translocation, inversion, duplication, and loss of several protein-coding genes in their genomes. The remaining cp genomes (Papaveroideae, Ranunculaceae, Berberidaceae, Menispermaceae, and Cicaeasteraceae) in the Ranunculales are highly conserved. The *accD* and *ndh* gene loss likely provides a prominent synapomorphic characteristic of the genus *Corydalis*. Phylogenetic and molecular clock studies offer new insights into the systematic relationships between *Corydalis* and will serve as a basis for future research on the phylogenetic, evolution, and biogeography relationships of *Corydalis* species.

## Data availability statement

The data presented in the study are deposited in the GenBank repository, accession number OP142703.

## Author contributions

GR, SP, and G-HN conceived the project. G-HN provided plant sources. GR designed the experiments. SP and G-HN supervised the project. GR performed the experiments, and analyzed the data, interpreted the results, and wrote and revised the manuscript. All authors read and approved the final manuscript.

## Supplementary material

Supplementary Table 1 List of taxa and GenBank accession numbers used in the phylogenetic and molecular clock analyses.

Supplementary Table 2 List of genes present in the chloroplast genome of *Corydalis platycarpa*.

Supplementary Table 3 Summary of the total genome size, GC content, LSC, SSC, and IR regions length, and gene content of 21 *Corydalis* cp genomes.

Supplementary Table 4 Distribution of distinct types of repeats in the 21 *Corydalis* cp genomes.

Supplementary Table 5 Presence of RNA editing sites, codon position, and amino acid conversion in the protein-coding genes of the 21 *Corydalis* plastomes.

Supplementary Table 6 Amount of synonymous and non-synonymous substitution rates present in the 59 protein-coding genes of the 21 *Corydalis* plastomes.

Supplementary Table 7 Amount of synonymous and non-synonymous substitution rates present in the 59 protein-coding genes of all the Ranunculales plastomes (37 plastomes).

Supplementary Table 8 Amount of synonymous and non-synonymous substitution rates present in the 59 protein-coding genes of all the Ranunculales (16 taxa) except the genus *Corydalis* plastomes.

Supplementary Table 9 Comparison of site models, positive selective amino acid loci, and estimation of parameters for 24 protein-coding genes in the *Corydalis* species.

Supplementary Table 10 List of pseudogenes and lost genes in the 21 *Corydalis* plastomes.

Supplementary Table 11 List of *Corydalis* plants distribution areas.

Supplementary Figure 1 MAUVE alignment of Ranunculales plastomes using Geneious Prime. Local collinear blocks are represented by blocks of the same color and linked within each of the alignments.

Supplementary Figure 2 Selective pressure analysis for 59 protein-coding genes of all the Ranunculales plastomes (37 taxa). **(A)** K_S_: rate of synonymous substitution; **(B)** K_A_: rate of non-synonymous substitution; **(C)** K_A_/K_S_: rate of non-synonymous vs. synonymous substitution.

Supplementary Figure 3 Selective pressure analysis for 59 protein-coding genes of all the Ranunculales plastomes (16 taxa) except *Corydalis* taxa. **(A)** K_S_: rate of synonymous substitution; **(B)** K_A_: rate of non-synonymous substitution; **(C)** K_A_/K_S_: rate of non-synonymous vs. synonymous substitution.

Supplementary Figure 4 Comparison of *clpP* gene in the *Corydalis platycarpa*, *C. fangshanensis*, *C. saxicola* with *C. adunca* plastome.

Supplementary Figure 5 Comparison of *psaI* gene in the LSC and IR region of *Corydalis platycarpa* plastome with LSC copy of *C. tomentela psaI*.
