# Supplementary material for: Extensive reorganization of the chloroplast genome of Corydalis platycarpa: A comparative analysis of their organization and evolution with other Corydalis plastomes
Source: Front Plant Sci. 2022 Dec 9;13:1043740. doi: 10.3389/fpls.2022.1043740 (PMC10115153; doi:10.3389/fpls.2022.1043740)
Supplement: Supplementary Table 1 — List of taxa and GenBank accession numbers used in the phylogenetic and molecular clock analyses. [file DataSheet_1.zip › Data Sheet 1/Supplementary Table S2.docx]

Supplementary Table S2. List of genes present in the chloroplast genome of *Corydalis platycarpa*.

| Category | Group of genes | Name of genes | | | | |
| --- | --- | --- | --- | --- | --- | --- |
| RNA genes | Ribosomal RNA genes | *rrn4.5*^a^ | *rrn5*^a^ | *rrn16*^a^ | *rrn23*^a^ |  |
|  | Transfer RNA genes | *trnA*-UGC^a, b^ | *trnC*-GCA | *trnD*-GUC | *trnE*-UUC | *trnF*-GAA |
|  |  | *trnfM*-CAU | *trnG*-GCC | *trnG*-UCC^a^ | *trnH*-GUG | *trnI*-CAU^a^ |
|  |  | *trnI*-GAU^a, b^ | *trnK*-UUU | *trnL*-CAA^a^ | *trnL*-UAA^b^ | *trnL*-UAG |
|  |  | *trnM*-CAU | *trnN*-GUU^a^ | *trnP*-UGG | *trnQ*-UUG | *trnR*-ACG^a^ |
|  |  | *trnR*-UCU | *trnS*-GCU | *trnS*-GGA | *trnS*-UGA | *trnT*-GGU |
|  |  | *trnT*-UGU | *trnV*-GAC^a^ | *trnV*-UAC^b^ | *trnW*-CCA | *trnY*-GUA |
| Photosynthetic genes | Subunits of photosystem I | *psaA* | *psaB* | *psaC*^a^ | *psaI* | *psaJ* |
|  |  | *ycf3*^c^ | *ycf4* |  |  |  |
|  | Subunits of photosystem II | *psbA* | *psbB* | *psbC* | *psbD* | *psbE* |
|  |  | *psbF* | *psbH* | *psbI*^a^ | *psbJ* | *psbK* |
|  |  | *psbL* | *psbM* | *psbN* | *psbT* | *psbZ* |
|  | Subunits of cytochrome | *petA* | *petB*^b^ | *petD*^b^ | *petG* | *petL* |
|  |  | *petN* |  |  |  |  |
|  | Subunits of ATP synthase | *atpA* | *atpB* | *atpE* | *atpF*^b^ | *atpH* |
|  |  | *atpI* |  |  |  |  |
|  | The large subunit of Rubisco | *rbcL* |  |  |  |  |
|  | Subunits of NADH dehydrogenase | *ndhA*^b^ | *ndhB*^a, b^ | *ndhC* | *ndhD*^a^ | *ndhE*^a^ |
|  |  | *ndhF*^a^ | *ndhG*^a^ | *ndhH* | *ndhI*^a^ | *ndhJ* |
|  |  | *ndhK* |  |  |  |  |
|  | ATP-dependent protease subunit P | *clpP*^d^ |  |  |  |  |
|  | Chloroplast envelope membrane protein | *cemA* |  |  |  |  |
| Transcription and translation-related genes | Small subunit of the ribosome | *rps2* | *rps3* | *rps4* | *rps7*^a^ | *rps8* |
|  |  | *rps11* | *rps12*^a, c, e^ | *rps14* | *rps15* | *rps16*^f^ |
|  |  | *rps18* | *rps19*^f^ |  |  |  |
|  | Large subunit of ribosome | *rpl2*^a, f^ | *rpl14* | *rpl16*^b^ | *rpl20* | *rpl22* |
|  |  | *rpl23*^a^ | *rpl32*^a^ | *rpl33* | *rpl36* |  |
|  | DNA-dependent RNA polymerase | *rpoA* | *rpoB* | *rpoC1*^b^ | *rpoC2* |  |
|  | Translation initiation factor | *infA* |  |  |  |  |
| Other genes | Maturase | *matK* |  |  |  |  |
|  | C-type cytochrome synthesis gene | *ccsA*^a^ |  |  |  |  |
|  | Component of TIC complex | *ycf1* |  |  |  |  |
| Genes of unknown function | Hypothetical proteins | *ycf2*^a^ |  |  |  |  |

^a^ - Two gene copies in IRs; ^b^ - Gene containing a single intron; ^c^ - Gene containing two introns; ^d^ - Gene containing three introns; ; ^e^ - Gene divided into two independent transcription units; ^f^ – Two copies – One intact gene and another is a pseudogene.
