# Supplementary material for: Extensive reorganization of the chloroplast genome of Corydalis platycarpa: A comparative analysis of their organization and evolution with other Corydalis plastomes
Source: Front Plant Sci. 2022 Dec 9;13:1043740. doi: 10.3389/fpls.2022.1043740 (PMC10115153; doi:10.3389/fpls.2022.1043740)
Supplement: Supplementary Table 1 — List of taxa and GenBank accession numbers used in the phylogenetic and molecular clock analyses. [file DataSheet_1.zip › Data Sheet 1/Supplementary Table S10.docx]

**Supplementary Table S10.** The list of pseudogenes and lost genes in the 21 *Corydalis* plastomes.

| Species | *accD* | *clpP*  (Int/Ex) | *ndhA* | *ndhB* | *ndhC* | *ndhD* | *ndhE* | *ndhF* | *ndhG* | *ndhH* | *ndhI* | *ndhJ* | *ndhK* | *rps16* | *psaI* | *trnV-UAC* |
| --- | --- | --- | --- | --- | --- | --- | --- | --- | --- | --- | --- | --- | --- | --- | --- | --- |
| *C. adunca* | - | + (2/3) | ψ | ψ | ψ | ψ | + | ψ | + | ψ | ψ | + | + | ++ | + | + |
| *C. conspersa* | - | - | - | - | - | ψ | ψ | - | + | ψ | ψ | - | - | + | + | + |
| *C. davidii* | - | + (2/3) | - | - | - | ψ | ψ | - | - | - | - | - | - | + | + | + |
| *C. edulis* | ψ | ψ | + | + | + | + | + | + | + | + | + | + | + | + | + | - |
| *C. fangshanensis* | - | + (3/4) | + | + | + | + | + | + | + | + | + | + | + | + | + | + |
| *C. filistipes* | - | - | + | + | + | + | + | + | + | + | + | + | + | + | + | + |
| *C. hsiaowutaishanensis* | - | - | + | + | + | + | + | + | + | + | + | + | + | + | + | + |
| *C. impatiens* | - | - | - | ψ | ψ | ψ | + | - | + | - | + | - | ψ | + | + | + |
| *C. inopinata* | - | + (2/3) | ψ | ψ | ψ | ψ | + | ψ | ψ | ψ | - | - | ψ | + | + | - |
| *C. lupinoides* | - | + (2/3) | ψ | - | - | ψ | ψ | ψ | ψ | ψ | - | - | - | + | + | - |
| *C. maculata* | - | - | + | + | + | + | + | + | + | + | + | + | + | + | + | + |
| *C. mucronifera* | - | - | + | - | - | + | + | - | + | + | + | ψ | - | + | + | + |
| *C. namdoensis* | - | - | + | + | + | + | + | + | + | + | + | + | + | + | + | + |
| *C. pauciovulata* | - | - | - | ψ | ψ | ψ | ψ | - | - | ψ | - | ψ | ψ | + | + | - |
| *C. platycarpa* | - | + (3/4) | + | + | + | + | + | + | + | + | + | + | + | + | ++ | + |
| *C. saxicola* | - | + (3/4) | + | + | + | + | + | + | + | + | + | + | + | + | ++ | + |
| *C. shensiana* | ψ | + (2/3) | + | + | + | + | + | + | + | + | + | + | + | + | + | - |
| *C. ternata* | - | - | + | + | + | + | + | + | + | + | + | + | + | ψ | - | + |
| *C. tomentella* | - | - | + | + | + | + | + | + | + | + | + | + | + | + | ++ | - |
| *C. trisecta* | ψ | + (2/3) | + | + | ψ | + | + | ψ | + | + | + | + | ψ | + | + | - |
| *C. turtschaninovii* | - | - | + | + | + | + | + | + | + | + | + | + | + | + | ++ | + |
