# Supplementary material for: Extensive reorganization of the chloroplast genome of Corydalis platycarpa: A comparative analysis of their organization and evolution with other Corydalis plastomes
Source: Front Plant Sci. 2022 Dec 9;13:1043740. doi: 10.3389/fpls.2022.1043740 (PMC10115153; doi:10.3389/fpls.2022.1043740)
Supplement: Supplementary Table 1 — List of taxa and GenBank accession numbers used in the phylogenetic and molecular clock analyses. [file DataSheet_1.zip › Data Sheet 1/Supplementary Table S9.docx]

**Supplementary Table S9.** Comparison of site models, positive selective amino acid loci and estimation of parameters for 24 protein-coding genes in the *Corydalis* species.

| Protein-coding gene | Model | np | Ln L | **Estimates of parameters** | | | | Model compared | LRT  P-value | Positive sites |
| --- | --- | --- | --- | --- | --- | --- | --- | --- | --- | --- |
| *atpB* | M3 | 45 | - 3851.29 | p: | 0.81648 | 0.01776 | 0.16577 | M0 vs. M3 | 0 | [] |
|  |  |  |  | ω: | 0 | 0 | 0.70014 |  |  |  |
|  | M0 | 41 | -3883.65 | ω0: | 0.10861 | | |  |  | Not Allowed |
|  | M2a | 44 | -3852.29 | p: | 0.88385 | 0.05773 | 0.05842 | M1a vs. M2a | 1.0 | [] |
|  |  |  |  | ω: | 0.01407 | 1.0 | 1.0 |  |  |  |
|  | M1a | 42 | -3852.29 | p: | 0.88385 | 0.11615 |  |  |  | Not Allowed |
|  |  |  |  | ω: | 0.01407 | 1.0 |  |  |  |  |
|  | M8 | 44 | -3852.23 | p0=0.88676 | p=0.03371 | q=0.33405 | | M7 vs. M8 | 0.00159 | 2 K 0.521,3 I 0.666,7 P 0.905,8 S 0.704,11 G 0.682,45 N 0.691,52 R 0.700,55 A 0.671,104 R 0.527,127 R 0.697,131 P 0.665,382 Q 0.676,445 S 0.605,456 R 0.680,468 V 0.785,485 A 0.680 |
|  |  |  |  | (p1=0.11324) | ω= 1.0 | | |  |  | Not Allowed |
|  | M7 | 42 | -3858.68 | p= | 0.03871 | q= | 0.21157 |  |  |  |
|  | M8a | 43 | -3851.59 | p0=093987 | p=0.03144 | q=0.45126 | | M8a vs. M8 | 0.254355 | Not Allowed |
|  |  |  |  | (p1= 0.0601) | ω= 1.00000 | | |  |  |  |
| *atpE* | M3 | 45 | -1151.71 | p: | 0.68691 | 0.20678 | 0.10632 | M0 vs. M3 | 0.077801 | [] |
|  |  |  |  | ω: | 0.10017 | 0.10017 | 0.84461 |  |  |  |
|  | M0 | 41 | -1155.92 | ω0: | 0.16780 | | |  |  | Not Allowed |
|  | M2a | 44 | -1151.76 | p: | 0.91488 | 0.03948 | 0.04564 | M1a vs. M2a | 1.0 | [] |
|  |  |  |  | ω: | 0.10750 | 1.0 | 1.0 |  |  |  |
|  | M1a | 42 | -1151.76 | p: | 0.91488 | 0.08512 |  |  |  | Not Allowed |
|  |  |  |  | ω: | 0.10750 | 1.0 |  |  |  |  |
|  | M8 | 44 | -1151.77 | p0=0.91611 | p=12.16902 | q=99.0 | | M7 vs. M8 | 0.495279 | 125 V 0.670,129 D 0.533 |
|  |  |  |  | (p1= 0.0839) | ω= 1.0 | | |  |  | Not Allowed |
|  | M7 | 42 | -1152.47 | p= | 0.036914 | q= | 1.49236 |  |  |  |
|  | M8a | 43 | -1151.78 | p0=0.91662 | p=12.15031 | q=99.0 | | M8a vs.M8 | 0.922518 | Not Allowed |
|  |  |  |  | (p1= 0.0834) | ω= 1.00000 | | |  |  |  |
| *atpF* | M3 | 45 | -1790.80 | p: | 0.53280 | 0.45871 | 0.00849 | M0 vs. M3 | 0.000836 | [] |
|  |  |  |  | ω: | 0.09950 | 0.85847 | 5.95252 |  |  |  |
|  | M0 | 41 | -1800.23 | ω0: | 0.44716 | | |  |  | Not Allowed |
|  | M2a | 44 | -1790.85 | p: | 0.61960 | 0.37330 | 0.00710 | M1a vs. M2a | 0.442999 | [] |
|  |  |  |  | ω: | 0.14281 | 1.0 | 6.45305 |  |  |  |
|  | M1a | 42 | -1791.67 | p: | 0.62270 | 0.37730 |  |  |  | Not Allowed |
|  |  |  |  | ω: | 0.13427 | 1.0 |  |  |  |  |
|  | M8 | 44 | -1790.85 | p0=0.99115 | p=17.15120 | q=18.82034 | | M7 vs.M8 | 0.353521 | 59 R 0.550,86 G 0.917,105 R 0.681,108 L 0.662 |
|  |  |  |  | (p1= 0.0089) | ω= 5.75340 | | |  |  | Not Allowed |
|  | M7 | 42 | -1791.89 | p= | 0.08921 | q= | 0.09681 |  |  |  |
|  | M8a | 43 | -1791.67 | p0=0.62429 | p=15.63372 | q=99.00 | | M8a vs.M8 | 0.200150 | Not Allowed |
|  |  |  |  | (p1= 0.3757) | ω= 1.00000 | | |  |  |  |
| *ccsA* | M3 | 45 | -1948.72 | p: | 0.71672 | 0.27933 | 0.00395 | M0 vs. M3 | 0.000001 | [] |
|  |  |  |  | ω: | 0 | 1.42394 | 14.39861 |  |  |  |
|  | M0 | 41 | -1965.25 | ω0: | 0.40873 | | |  |  | Not Allowed |
|  | M2a | 44 | -1949.29 | p: | 0.66312 | 0.32893 | 0.00795 | M1a vs. M2a | 0.064451 | [] |
|  |  |  |  | ω: | 0 | 1.0 | 8.48850 |  |  |  |
|  | M1a | 42 | -1952.03 | p: | 0.67699 | 0.32301 |  |  |  | Not Allowed |
|  |  |  |  | ω: | 0 | 1.0 |  |  |  |  |
|  | M8 | 44 | -1949.29 | p0=0.99254 | p=0.00500 | q=0.00879 | | M7 vs.M8 | 0.063410 | 23 T 0.758,39 Y 0.625,72 L 0.607,76 L 0.634,88 M 0.560,120 A 0.738,176 R 0.710,179 V 0.891,188 S 0.758,231 L 0.786,278 Q 0.742,280 F 0.553,298 I 0.595,318 L 0.992** |
|  |  |  |  | (p1= 0.0075) | ω= 8.81041 | | |  |  | Not Allowed |
|  | M7 | 42 | -1952.05 | p= | 0.01234 | q= | 0.02704 |  |  |  |
|  | M8a | 43 | -1952.03 | p0=0.67699 | p=00500 | q=3.03477 | | M8a vs.M8 | 0.019199 | Not Allowed |
|  |  |  |  | (p1=0.32301) | ω= 1.00000 | | |  |  |  |
| *matK* | M3 | 45 | -3415.33 | p: | 0.46630 | 0.51071 | 0.02298 | M0 vs. M3 | 0 | [] |
|  |  |  |  | ω: | 0.06817 | 0.76319 | 3.83899 |  |  |  |
|  | M0 | 41 | -3440.45 | ω0: | 0.46672 | | |  |  | Not Allowed |
|  | M2a | 44 | -3415.65 | p: | 0.61161 | 0.37305 | 0.01534 | M1a vs. M2a | 0.139655 | [] |
|  |  |  |  | ω: | 0.14025 | 1.0 | 4.38947 |  |  |  |
|  | M1a | 42 | -3417.62 | p: | 0.61346 | 0.38654 |  |  |  | Not Allowed |
|  |  |  |  | ω: | 0.12785 | 1.0 |  |  |  |  |
|  | M8 | 44 | -3415.41 | p0=0.97923 | p=0.37612 | q=0.48236 | | M7 vs.M8 | 0.070501 | 40 Y 0.563,81 P 0.886,127 I 0.914,141 A 0.751,148 P 0.660,163 R 0.647,177 A 0.625,301 R 0.814,303 Q 0.934 |
|  |  |  |  | (p1=0.02077) | ω=3.93093 | | |  |  | Not Allowed |
|  | M7 | 42 | -3418.06 | p= | 0.08366 | q= | 0.09095 |  |  |  |
|  | M8a | 43 | 3417.62 | p0=0.61533 | p=14.79416 | q=8.96718 | | M8a vs.M8 | 0.035405 | Not Allowed |
|  |  |  |  | (p1=0.38467) | ω= 1.00000 | | |  |  |  |
| *psbH* | M3 | 45 | -600.501 | p: | 0.65190 | 0.34147 | 0.00664 | M0 vs. M3 | 0.122044 | [] |
|  |  |  |  | ω: | 0 | 0.49155 | 3.45175 |  |  |  |
|  | M0 | 41 | -604.139 | ω0: | 0.17760 | | |  |  | Not Allowed |
|  | M2a | 44 | -601.302 | p: | 0.82974 | 0.08369 | 0.08658 | M1a vs. M2a | 1.0 | [] |
|  |  |  |  | ω: | 0.05048 | 1.0 | 1.0 |  |  |  |
|  | M1a | 42 | -601.302 | p: | 0.82974 | 0.17026 |  |  |  | Not Allowed |
|  |  |  |  | ω: | 0.05048 | 1.0 |  |  |  |  |
|  | M8 | 44 | -600.504 | p0=0.99386 | p=0.03784 | q=0.18425 | | M7 vs. M8 | 0.949769 | 45 V 0.782 |
|  |  |  |  | (p1=0.00614) | ω=3.44353 | | |  |  | Not Allowed |
|  | M7 | 42 | -600.555 | p= | 0.04098 | q= | 0.19231 |  |  |  |
|  | M8a | 43 | -600.778 | p0=0.99999 | p=0.15844 | q=0.69014 | | M8a vs. M8 | 0.459456 | Not Allowed |
|  |  |  |  | (p1=0.00001) | ω= 1.00000 | | |  |  |  |
| *psbJ* | M3 | 45 | -319.122 | p: | 0.72726 | 0.24834 | 0.02440 | M0 vs. M3 | 0 | [] |
|  |  |  |  | ω: | 0 | 0.68204 | 46.71221 |  |  |  |
|  | M0 | 41 | -350.042 | ω0: | 0.05685 | | |  |  | Not Allowed |
|  | M2a | 44 | -319.211 | p: | 0.81049 | 0.16510 | 0.02440 | M1a vs. M2a | 0 | [] |
|  |  |  |  | ω: | 0.02621 | 1.0 | 47.83630 |  |  |  |
|  | M1a | 42 | -349.516 | p: | 0.95528 | 0.04472 |  |  |  | Not Allowed |
|  |  |  |  | ω: | 0.03587 | 1.0 |  |  |  |  |
|  | M8 | 44 | -319.200 | p0=0.97533 | p=0.00548 | q=0.02733 | | M7 vs. M8 | 0 | 41 - 1.000** |
|  |  |  |  | (p1= 0.0245) | ω=47.1691 | | |  |  | Not Allowed |
|  | M7 | 42 | -349.090 | p= | 0.02624 | q= | 0.16055 |  |  |  |
|  | M8a | 43 | -349.078 | p0=0.9999 | p=0.15322 | q=1.94149 | | M8a vs.M8 | 0 | Not Allowed |
|  |  |  |  | (p1=0.00001) | ω= 1.00000 | | |  |  |  |
| *psbK* | M3 | 45 | -516.927 | p: | 0.98378 | 0.0007 | 0.01615 | M0 vs. M3 | 0 | [] |
|  |  |  |  | ω: | 0.45579 | 0.45580 | 64.54044 |  |  |  |
|  | M0 | 41 | -549.257 | ω0: | 0.19339 | | |  |  | Not Allowed |
|  | M2a | 44 | -516.927 | p: | 0.98385 | 0 | 0.01615 | M1a vs. M2a | 0 | [] |
|  |  |  |  | ω: | 0.45579 | 1.0 | 64.54034 |  |  |  |
|  | M1a | 42 | -549.257 | p: | 0.99999 | 0.00001 |  |  |  | Not Allowed |
|  |  |  |  | ω: | 0.19339 | 1.0 |  |  |  |  |
|  | M8 | 44 | -516.936 | p0=0.98385 | p=83.36816 | q=99.0 | | M7 vs.M8 | 0 | 62 - 1.000** |
|  |  |  |  | (p1= 0. 0162) | ω=64.6378 | | |  |  | Not Allowed |
|  | M7 | 42 | -549.304 | p= | 24.00653 | q= | 99.0 |  |  |  |
|  | M8a | 43 | -549.323 | p0=0.99999 | p=24.1417 | q=99.00 | | M8a vs.M8 | 0 | Not Allowed |
|  |  |  |  | (p1= 0.0001) | ω= 1.00000 | | |  |  |  |
| *psbT* | M3 | 45 | -288.218 | p: | 0.57277 | 0.36023 | 0.06700 | M0 vs. M3 | 0.741415 | [] |
|  |  |  |  | ω: | 0.01707 | 0.71470 | 0.71470 |  |  |  |
|  | M0 | 41 | -289.203 | ω0: | 0.31042 | | |  |  | Not Allowed |
|  | M2a | 44 | -288.273 | p: | 0.74336 | 0.15483 | 0.10181 | M1a vs. M2a | 1.0 | [] |
|  |  |  |  | ω: | 0.09823 | 1.0 | 1.0 |  |  |  |
|  | M1a | 42 | -288.273 | p: | 0.74336 | 025664 |  |  |  | Not Allowed |
|  |  |  |  | ω: | 0.09823 | 1.0 |  |  |  |  |
|  | M8 | 44 | -288.230 | p0=0.96981 | p=0.21707 | q=0.51988 | | M7 vs.M8 | 0.999047 | -- |
|  |  |  |  | (p1= 0.0302) | ω=1.0 | | |  |  | Not Allowed |
|  | M7 | 42 | -288.231 | p= | 0.22384 | q= | 0.49314 |  |  |  |
|  | M8a | 43 | -288.245 | p0=0.99999 | p=0.21268 | q=0.45442 | | M8a vs.M8 | 0.861662 | Not Allowed |
|  |  |  |  | (p1=0.00001) | ω= 1.00000 | | |  |  |  |
| *rpl16* | M3 | 45 | -1172.52 | p: | 0.47780 | 0.49479 | 0.02741 | M0 vs. M3 | 0.013888 | [] |
|  |  |  |  | ω: | 0 | 0.19504 | 1.53700 |  |  |  |
|  | M0 | 41 | -1178.78 | ω0: | 0.13227 | | |  |  | Not Allowed |
|  | M2a | 44 | 1172.70 | p: | 0.92786 | 0.03870 | 0.03344 | M1a vs. M2a | 1.0 | [] |
|  |  |  |  | ω: | 0.07246 | 1.0 | 1.0 |  |  |  |
|  | M1a | 42 | -1172.70 | p: | 0.92786 | 0.07214 |  |  |  | Not Allowed |
|  |  |  |  | ω: | 0.07246 | 1.0 |  |  |  |  |
|  | M8 | 44 | -1172.52 | p0=0.97495 | p=0.39908 | q=2.95554 | | M7 vs.M8 | 0.296126 | 21 Y 0.892,120 A 0.627 |
|  |  |  |  | (p1=0.02505) | ω= 1.58581 | | |  |  | Not Allowed |
|  | M7 | 42 | 1173.75 | p= | 0.19476 | q= | 1.03439 |  |  |  |
|  | M8a | 43 | -1172.63 | p0=0.94118 | p=1.05562 | q=11.58846 | | M8a vs.M8 | 0.662062 | Not Allowed |
|  |  |  |  | (p1=0.05882) | ω= 1.00000 | | |  |  |  |
| *rpl20* | M3 | 45 | -1203.79 | p: | 0.47373 | 0.44263 | 0.08364 | M0 vs. M3 | 0 | [] |
|  |  |  |  | ω: | 0 | 0.56290 | 4.06602 |  |  |  |
|  | M0 | 41 | -1244.94 | ω0: | 0.46621 | | |  |  | Not Allowed |
|  | M2a | 44 | -1205.27 | p: | 0.62701 | 0.30685 | 0.06614 | M1a vs. M2a | 0.000042 | [] |
|  |  |  |  | ω: | 0.05554 | 1.0 | 4.88408 |  |  |  |
|  | M1a | 42 | -1215.35 | p: | 0.66897 | 0.33103 |  |  |  | Not Allowed |
|  |  |  |  | ω: | 0.05401 | 1.0 |  |  |  |  |
|  | M8 | 44 | -1204.37 | p0=0.91919 | p=0.25201 | q=0.65410 | | M7 vs. M8 | 0.000020 | 29 S 0.900,73 A 0.887,75 Y 0.710,79 R 0.633,91 S 0.573,93 R 0.678,102 E 0.983*,104 Q 1.000**,105 F 0.972* |
|  |  |  |  | (p1= 0.0808) | ω=4.10312 | | |  |  | Not Allowed |
|  | M7 | 42 | -1215.17 | p= | 0.05790 | q= | 0.07224 |  |  |  |
|  | M8a | 43 | -1215.09 | p0=0.71025 | p=0.36680 | q=3.77745 | | M8a vs. M8 | 0.000004 | Not Allowed |
|  |  |  |  | (p1= 0.2898) | ω= 1.00000 | | |  |  |  |
| *rpl22* | M3 | 45 | -1660.95 | p: | 0.59834 | 0.30050 | 0.10116 | M0 vs. M3 | 0 | [] |
|  |  |  |  | ω: | 0.07309 | 0.72479 | 3.52794 |  |  |  |
|  | M0 | 41 | -1722.68 | ω0: | 0.48681 | | |  |  | Not Allowed |
|  | M2a | 44 | -1661.04 | p: | 0.68280 | 0.23530 | 0.08190 | M1a vs. M2a | 0.000002 | [] |
|  |  |  |  | ω: | 0.10643 | 1.0 | 3.97669 |  |  |  |
|  | M1a | 42 | -1674.06 | p: | 0.70613 | 0.29387 |  |  |  | Not Allowed |
|  |  |  |  | ω: | 0.08459 | 1.0 |  |  |  |  |
|  | M8 | 44 | -1660.90 | p0=0.89462 | p=0.32564 | q=0.78400 | | M7 vs. M8 | 0.000001 | 6 Q 0.627,7 G 0.530,13 L 0.795,18 R 0.897,37 T 0.999**,41 L 0.999**,44 K 0.891,45 L 0.980*,52 V 0.987*,55 L 0.996**,70 R 0.520,117 P 0.819,121 W 0.953*,122 K 0.773 |
|  |  |  |  | (p1= 0.1054) | ω=3.4511 | | |  |  | Not Allowed |
|  | M7 | 42 | -1674.59 | p= | 0.06309 | q= | 0.07699 |  |  |  |
|  | M8a | 43 | -1673.98 | p0=0.71653 | p=1.91087 | q=18.70185 | | M8a vs.M8 | 0.000003 | Not Allowed |
|  |  |  |  | (p1= 0.2835) | ω= 1.00000 | | |  |  |  |
| *rpl23* | M3 | 45 | -850.370 | p: | 0.52771 | 0.44571 | 0.02658 | M0 vs. M3 | 0.000008 | [] |
|  |  |  |  | ω: | 0 | 0.54921 | 4.04160 |  |  |  |
|  | M0 | 41 | -864.782 | ω0: | 0.29174 | | |  |  | Not Allowed |
|  | M2a | 44 | -850.821 | p: | 0.73054 | 0.25622 | 0.01324 | M1a vs. M2a | 0.194723 | [] |
|  |  |  |  | ω: | 0.07250 | 1.0 | 6.90442 |  |  |  |
|  | M1a | 42 | -852.457 | p: | 0.73489 | 0.26511 |  |  |  | Not Allowed |
|  |  |  |  | ω: | 0.06832 | 1.0 |  |  |  |  |
|  | M8 | 44 | -850.403 | p0=0.98279 | p=0.21396 | q=0.56896 | | M7 vs.M8 | 0.108094 | 27 S 0.666,78 L 0.971* |
|  |  |  |  | (p1= 0.0172) | ω=5.4220 | | |  |  | Not Allowed |
|  | M7 | 42 | -852.628 | p= | 0.16586 | q= | 0.34689 |  |  |  |
|  | M8a | 43 | -852.265 | p0=0.79307 | p=0.36847 | q=2.77203 | | M8a vs.M8 | 0.053639 | Not Allowed |
|  |  |  |  | (p1= 0.2069) | ω= 1.00000 | | |  |  |  |
| *rpl33* | M3 | 45 | -576.766 | p: | 0.78602 | 0.14147 | 0.07251 | M0 vs. M3 | 0.009551 | [] |
|  |  |  |  | ω: | 0.10252 | 1.17674 | 1.17675 |  |  |  |
|  | M0 | 41 | -583.457 | ω0: | 0.25072 | | |  |  | Not Allowed |
|  | M2a | 44 | -576.766 | p: | 0.78602 | 0 | 0.21398 | M1a vs. M2a | 0.942989 | [] |
|  |  |  |  | ω: | 0.10252 | 1.0 | 1.17674 |  |  |  |
|  | M1a | 42 | -576.825 | p: | 0.76298 | 0.23702 |  |  |  | Not Allowed |
|  |  |  |  | ω: | 0.09237 | 1.0 |  |  |  |  |
|  | M8 | 44 | -576.779 | p0=0.78819 | p=11.6355 | q=99.0 | | M7 vs.M8 | 0.454215 | 4 G 0.712,6 V 0.521,19 R 0.509 |
|  |  |  |  | (p1= 0.2118) | ω= 1.18114 | | |  |  | Not Allowed |
|  | M7 | 42 | -577.568 | p= | 0.24667 | q= | 0.53926 |  |  |  |
|  | M8a | 43 | -576.846 | p0=0.76526 | p=10.3633 | q=99.0 | | M8a vs.M8 | 0.714526 | Not Allowed |
|  |  |  |  | (p1=0.23474) | ω= 1.00000 | | |  |  |  |
| *rps2* | M3 | 45 | -1978.01 | p: | 0.08007 | 0.82278 | 0.09715 | M0 vs. M3 | 0 | [] |
|  |  |  |  | ω: | 0.21952 | 0.21952 | 2.97148 |  |  |  |
|  | M0 | 41 | -2006.27 | ω0: | 0.43980 | | |  |  | Not Allowed |
|  | M2a | 44 | -1978.01 | p: | 0.90285 | 0 | 0.09715 | M1a vs. M2a | 0.001145 | [] |
|  |  |  |  | ω: | 0.21952 | 1.0 | 2.97149 |  |  |  |
|  | M1a | 42 | -1984.77 | p: | 0.72156 | 0.27844 |  |  |  | Not Allowed |
|  |  |  |  | ω: | 0.09186 | 1.0 |  |  |  |  |
|  | M8 | 44 | -1978.01 | p0=0.90328 | p=28.05851 | q=99.0 | | M7 vs.M8 | 0.000684 | 23 K 0.637,25 K 0.601,26 K 0.950,30 R 0.957*,42 M 0.512,70 Q 0.729,119 S 0.948,124 E 0.869,149 E 0.699,160 G 0.807,181 I 0.971*,191 I 0.827,212 G 0.877 |
|  |  |  |  | (p1= 0.0967) | ω=2.97850 | | |  |  | Not Allowed |
|  | M7 | 42 | -1985.30 | p= | 0.05891 | q= | 0.07313 |  |  |  |
|  | M8a | 43 | -1984.81 | p0=0.72136 | p=10.11768 | q=99.0 | | M8a vs.M8 | 0.000227 | Not Allowed |
|  |  |  |  | (p1=0.27864) | ω=1.0 | | |  |  |  |
| *rps3* | M3 | 45 | -2185.59 | p: | 0.81936 | 0.16214 | 0.01850 | M0 vs. M3 | 0 | [] |
|  |  |  |  | ω: | 0.21301 | 2.17050 | 11.31303 |  |  |  |
|  | M0 | 41 | -2246.05 | ω0: | 0.54947 | | |  |  | Not Allowed |
|  | M2a | 44 | -2188.40 | p: | 0.67494 | 0.26535 | 0.05971 | M1a vs. M2a | 0 | [] |
|  |  |  |  | ω: | 0.13380 | 1.0 | 5.24596 |  |  |  |
|  | M1a | 42 | -2206.34 | p: | 0.68215 | 0.31785 |  |  |  | Not Allowed |
|  |  |  |  | ω: | 0.09272 | 1.0 |  |  |  |  |
|  | M8 | 44 | -2188.76 | p0=0.92232 | p=0.39062 | q=0.71085 | | M7 vs.M8 | 0 | 54 I 0.933,64 I 0.923,70 I 0.897,100 E 0.999**,137 A 0.878,140 K 0.820,144 T 0.876,149 P 0.999**,151 K 1.000**,152 E 0.705,153 G 0.827,160 V 0.603,213 G 0.975* |
|  |  |  |  | (p1=0.0777) | ω=4.64056 | | |  |  | Not Allowed |
|  | M7 | 42 | -2207.13 | p= | 0.06263 | q= | 0.07533 |  |  |  |
|  | M8a | 43 | -2206.38 | p0=0.68298 | p=10.29730 | q=99.0 | | M8a vs.M8 | 0 | Not Allowed |
|  |  |  |  | (p1=0.3170) | ω=1.0 | | |  |  |  |
| *rps4* | M3 | 45 | 1906.77 | p: | 060848 | 0.31343 | 0.07810 | M0 vs. M3 | 0 | [] |
|  |  |  |  | ω: | 0 | 0.73340 | 4.83831 |  |  |  |
|  | M0 | 41 | -1988.89 | ω0: | 0.42372 | | |  |  | Not Allowed |
|  | M2a | 44 | -1907.17 | p: | 0.67496 | 0.25595 | 0.06909 | M1a vs. M2a | 0 | [] |
|  |  |  |  | ω: | 0.02265 | 1.0 | 5.28113 |  |  |  |
|  | M1a | 42 | -1926.01 | p: | 0.71116 | 0.28884 |  |  |  | Not Allowed |
|  |  |  |  | ω: | 0.02327 | 1.0 |  |  |  |  |
|  | M8 | 44 | -1906.77 | p0=0.92208 | p=0.03414 | q=0.14182 | | M7 vs.M8 | 0 | 13 K 0.906,23 R 0.984*,25 S 1.000**,26 P 0.994**,27 D 0.871,28 R 0.969*,31 R 0.771,32 K 0.556,33 K 0.998**,34 P 0.989*,37 R 0.991**,38 R 0.992**,67 S 0.702 |
|  |  |  |  | (p1=0.07792) | ω=4.84178 | | |  |  | Not Allowed |
|  | M7 | 42 | -1926.36 | p= | 0.03987 | q= | 0.05406 |  |  |  |
|  | M8a | 43 | -1926.01 | p0=0.71163 | p=2.44269 | q=99.0 | | M8a vs.M8 | 0 | Not Allowed |
|  |  |  |  | (p1=0.2884) | ω=1.0 | | |  |  |  |
| *rps7* | M3 | 45 | -1095.23 | p: | 0.84149 | 0.15209 | 0.00642 | M0 vs. M3 | 0 | [] |
|  |  |  |  | ω: | 0.24691 | 2.42615 | 140.5701 |  |  |  |
|  | M0 | 41 | -1156.44 | ω0: | 0.23751 | | |  |  | Not Allowed |
|  | M2a | 44 | -1097.60 | p: | 0.55251 | 0.44106 | 0.00643 | M1a vs. M2a | 0 | [] |
|  |  |  |  | ω: | 0.01602 | 1.0 | 107.1382 |  |  |  |
|  | M1a | 42 | -1153.80 | p: | 0.82293 | 0.17707 |  |  |  | Not Allowed |
|  |  |  |  | ω: | 0.11020 | 1.0 |  |  |  |  |
|  | M8 | 44 | -1096.13 | p0=0.99357 | p=0.07370 | q=0.08283 | | M7 vs.M8 | 0 | 131 R 0.799,156 - 1.000** |
|  |  |  |  | (p1=0.0064) | ω=119.065 | | |  |  | Not Allowed |
|  | M7 | 42 | -1153.99 | p= | 0.25391 | q= | 0.69562 |  |  |  |
|  | M8a | 43 | -1153.81 | p0=0.82431 | p=12.46444 | q=98.96507 | | M8a vs.M8 | 0 | Not Allowed |
|  |  |  |  | (p1=0.17569) | ω=1.0 | | |  |  |  |
| *rps8* | M3 | 45 | -1239.56 | p: | 0.77669 | 0.21585 | 0.00747 | M0 vs. M3 | 0 | [] |
|  |  |  |  | ω: | 0 | 1.26197 | 32.47013 |  |  |  |
|  | M0 | 41 | -1302.94 | ω0: | 0.17226 | | |  |  | Not Allowed |
|  | M2a | 44 | -1240.04 | p: | 0.76255 | 0.22998 | 0.00747 | M1a vs. M2a | 0 | [] |
|  |  |  |  | ω: | 0 | 1.0 | 30.29753 |  |  |  |
|  | M1a | 42 | -1277.82 | p: | 0.78789 | 0.21211 |  |  |  | Not Allowed |
|  |  |  |  | ω: | 0 | 1.0 |  |  |  |  |
|  | M8 | 44 | -1242.10 | p0=0.99253 | p=0.00500 | q=0.00707 | | M7 vs.M8 | 0 | 74 Y 0.674,134 - 1.000** |
|  |  |  |  | (p1=0.00747) | ω=32.3329 |  |  |  |  | Not Allowed |
|  | M7 | 42 | -1280.36 | p= | 0.01742 | q= | 0.07941 |  |  |  |
|  | M8a | 43 | -1277.82 | p0=0.78789 | p=0.00500 | q=14.42554 | | M8a vs.M8 | 0 | Not Allowed |
|  |  |  |  | (p1=0.21211) | ω=1.0 | | |  |  |  |
| *rps11* | M3 | 45 | -1509.53 | p: | 0.66193 | 0.31923 | 0.01884 | M0 vs. M3 | 0 | [] |
|  |  |  |  | ω: | 0.06865 | 1.05769 | 7.09393 |  |  |  |
|  | M0 | 41 | -1537.76 | ω0: | 0.40026 | | |  |  | Not Allowed |
|  | M2a | 44 | -1509.55 | p: | 0.64578 | 0.33504 | 0.01918 | M1a vs. M2a | 0.010488 | [] |
|  |  |  |  | ω: | 0.06125 | 1.0 | 7.00178 |  |  |  |
|  | M1a | 42 | -1514.11 | p: | 0.65664 | 0.34336 |  |  |  | Not Allowed |
|  |  |  |  | ω: | 0.05849 | 1.0 |  |  |  |  |
|  | M8 | 44 | -1509.59 | p0=0.98074 | p=0.06802 | q=0.08701 | | M7 vs.M8 | 0.010045 | 36 L 0.545,88 R 0.505,98 L 0.824,108 G 0.994**,116 L 0.961* |
|  |  |  |  | (p1=0.01926) | ω=6.98704 | | |  |  | Not Allowed |
|  | M7 | 42 | -1514.19 | p= | 0.06007 | q= | 0.07527 |  |  |  |
|  | M8a | 43 | -1514.11 | p0=0.65755 | p=6.30041 | q=99.0 | | M8a vs.M8 | 0.002636 | Not Allowed |
|  |  |  |  | (p1=0.34245) | ω=1.0 | | |  |  |  |
| *rps14* | M3 | 45 | -329.278 | p: | 0.79463 | 0.12464 | 0.08074 | M0 vs. M3 | 0.000015 | [] |
|  |  |  |  | ω: | 0 | 2.56727 | 2.56727 |  |  |  |
|  | M0 | 41 | -343.119 | ω0: | 0.43444 | | |  |  | Not Allowed |
|  | M2a | 44 | -329.278 | p: | 0.79463 | 0 | 0.20537 | M1a vs. M2a | 0.152229 | [] |
|  |  |  |  | ω: | 0 | 1.0 | 2.56727 |  |  |  |
|  | M1a | 42 | -331.160 | p: | 0.76591 | 0.23409 |  |  |  | Not Allowed |
|  |  |  |  | ω: | 0 | 1.0 |  |  |  |  |
|  | M8 | 44 | -329.278 | p0=0.79463 | p=0.00500 | q=1.51501 | | M7 vs.M8 | 0.080102 | 29 N 0.549,31 I 0.577,32 S 0.705,36 A 0.983*,37 M 0.971*,39 E 0.601,40 K 0.567,42 E 0.576 |
|  |  |  |  | (p1=0.20537) | ω=2.56726 | | |  |  | Not Allowed |
|  | M7 | 42 | -331.802 | p= | 0.00604 | q= | 0.01623 |  |  |  |
|  | M8a | 43 | -331.160 | p0=0.76591 | p=0.00500 | q=99.0 | | M8a vs.M8 | 0.052344 | Not Allowed |
|  |  |  |  | (p1=0.23409) | ω=1.0 | | |  |  |  |
| *rps15* | M3 | 45 | -552.115 | p: | 0.00399 | 0.98941 | 0.00659 | M0 vs. M3 | 1.0 | [] |
|  |  |  |  | ω: | 0.27482 | 0.27482 | 0.27482 |  |  |  |
|  | M0 | 41 | -552.115 | ω0: | 0.27482 | | |  |  | Not Allowed |
|  | M2a | 44 | -552.115 | p: | 1.0 | 0 | 0 | M1a vs. M2a | 0.999929 | [] |
|  |  |  |  | ω: | 0.27482 | 1.0 | 1.0 |  |  |  |
|  | M1a | 42 | -552.115 | p: | 0.99999 | 0.00001 |  |  |  | Not Allowed |
|  |  |  |  | ω: | 0.27482 | 1.0 |  |  |  |  |
|  | M8 | 44 | -552.146 | p0=0.99999 | p=38.12440 | q=99.0 | | M7 vs.M8 | 0.999916 | -- |
|  |  |  |  | (p1=0.00001) | ω=1.10374 |  |  |  |  | Not Allowed |
|  | M7 | 42 | -552.146 | p= | 38.10567 | q= | 99.0 |  |  |  |
|  | M8a | 43 | -552.159 | p0=0.99999 | P=38.2699 | q=99.0 | | M8a vs.M8 | 0.873044 | Not Allowed |
|  |  |  |  | (p1=0.00001) | ω=1.0 | | |  |  |  |
| *rps16* | M3 | 45 | -571.502 | p: | 0.11825 | 0.73889 | 0.14287 | M0 vs. M3 | 0.000049 | [] |
|  |  |  |  | ω: | 0.07404 | 0.86253 | 6.23668 |  |  |  |
|  | M0 | 41 | -586.492 | ω0: | 1.14579 | | |  |  | Not Allowed |
|  | M2a | 44 | -571.572 | p: | 0.15716 | 0.70399 | 0.13884 | M1a vs. M2a | 0.000036053 | [] |
|  |  |  |  | ω: | 0.12934 | 1.0 | 6.78413 |  |  |  |
|  | M1a | 42 | -581.587 | p: | 0.43366 | 0.56634 |  |  |  | Not Allowed |
|  |  |  |  | ω: | 0.22240 | 1.0 |  |  |  |  |
|  | M8 | 44 | -571.757 | p0=0.86039 | p=0.19275 | q=0.04948 | | M7 vs.M8 | 0.000045 | 13 H 0.999**,22 A 0.895,26 E 1.000**,32 V 0.995** |
|  |  |  |  | (p1=0.13961) | ω=6.26934 |  |  |  |  | Not Allowed |
|  | M7 | 42 | -581.711 | p= | 0.12370 | q= | 0.03090 |  |  |  |
|  | M8a | 43 | -581.547 | p0=0.47439 | p=3.61742 | q=10.43333 | | M8a vs.M8 | 0.000048 | Not Allowed |
|  |  |  |  | (p1=0.52561) | ω=1.0 | | |  |  |  |
| *rps18* | M3 | 45 | -872.793 | p: | 0.57399 | 0.11292 | 0.31308 | M0 vs. M3 | 0.000012 | [] |
|  |  |  |  | ω: | 0.38165 | 0.38165 | 2.43528 |  |  |  |
|  | M0 | 41 | -886.853 | ω0: | 0.87492 | | |  |  | Not Allowed |
|  | M2a | 44 | -872.793 | p: | 0.68692 | 0 | 0.31308 | M1a vs. M2a | 0.008383 | [] |
|  |  |  |  | ω: | 0.38165 | 1.0 | 2.43528 |  |  |  |
|  | M1a | 42 | -877.575 | p: | 0.51851 | 0.48149 |  |  |  | Not Allowed |
|  |  |  |  | ω: | 0.18630 | 1.0 |  |  |  |  |
|  | M8 | 44 | -872.799 | p0=0.68777 | p=61.58653 | q=99.0 | | M7 vs.M8 | 0.002888 | 7 F 0.925,9 S 0.907,15 R 0.757,17 L 0.788,18 P 0.908,19 S 0.985*,20 G 0.927,24 D 0.794,28 L 0.964*,29 S 0.754,35 I 0.579,38 D 0.890,49 L 0.506 |
|  |  |  |  | (p1=0.31223) | ω=2.43998 | | |  |  | Not Allowed |
|  | M7 | 42 | -878.647 | p= | 0.14376 | q= | 0.03896 |  |  |  |
|  | M8a | 43 | -877.619 | p0=0.51929 | p=23.11059 | q=99.0 | | M8a vs.M8 | 0.001905 | Not Allowed |
|  |  |  |  | (p1=0.48071) | ω=1.0 | | |  |  |  |

Note:

[] – No data available

np represents the degree of freedom

Positively selected sites (* *p* > 95%; ** *p* > 99%)
