# Supplementary material for: Extensive reorganization of the chloroplast genome of Corydalis platycarpa: A comparative analysis of their organization and evolution with other Corydalis plastomes
Source: Front Plant Sci. 2022 Dec 9;13:1043740. doi: 10.3389/fpls.2022.1043740 (PMC10115153; doi:10.3389/fpls.2022.1043740)
Supplement: Supplementary Table 1 — List of taxa and GenBank accession numbers used in the phylogenetic and molecular clock analyses. [file DataSheet_1.zip › Data Sheet 1/Supplementary Table S11.docx]

**Supplementary Table S11**: The list of *Corydalis* plants distribution areas

| S. N. | Species name | Plant distribution areas |
| --- | --- | --- |
|  | *Corydalis adunca* | China North-Central, China South-Central, Inner Mongolia, Qinghai, Tadzhikistan, Tibet |
|  | *Corydalis conspersa* | China North-Central, China South-Central, Nepal, Qinghai, Tibet |
|  | *Corydalis davidii* | Assam, China South-Central, Myanmar |
|  | *Corydalis edulis* | China North-Central, China South-Central, China Southeast, Manchuria |
|  | *Corydalis fangshanensis* | China North-Central, China Southeast |
|  | *Corydalis filistipes* | Korea |
|  | *Corydalis hsiaowutaishanensis* | China North-Central |
|  | *Corydalis impatiens* | Buryatiya, China North-Central, Chita, Inner Mongolia, Irkutsk, Manchuria, Mongolia, Qinghai, Tuva |
|  | *Corydalis inopinata* | East Himalaya, Tibet, West Himalaya |
|  | *Corydalis lupinoides* | Tibet |
|  | *Corydalis maculata* | Korea |
|  | *Corydalis mucronifera* | China North-Central, Qinghai, Tibet, Xinjiang |
|  | *Corydalis namdoensis* | South Korea (Korea, Eastern Asia, Asia-Temperate) |
|  | *Corydalis pauciovulata* | Japan, Korea |
|  | *Corydalis platycarpa* | Japan, Kazan-retto, Korea, Nansei-shoto, Ogasawara-shoto |
|  | *Corydalis saxicola* | China North-Central, China South-Central, China Southeast |
|  | *Corydalis shensiana* | China North-Central, China Southeast |
|  | *Corydalis ternata* | Japan, Korea, Manchuria |
|  | *Corydalis tomentella* | China North-Central, China South-Central |
|  | *Corydalis trisecta* | China North-Central, China South-Central, China Southeast |
|  | *Corydalis turtschaninovii* | Amur, China North-Central, Chita, Inner Mongolia, Japan, Khabarovsk, Korea, Manchuria, Primorye |
