# Supplementary material for: Extensive reorganization of the chloroplast genome of Corydalis platycarpa: A comparative analysis of their organization and evolution with other Corydalis plastomes
Source: Front Plant Sci. 2022 Dec 9;13:1043740. doi: 10.3389/fpls.2022.1043740 (PMC10115153; doi:10.3389/fpls.2022.1043740)
Supplement: Supplementary Table 1 — List of taxa and GenBank accession numbers used in the phylogenetic and molecular clock analyses. [file DataSheet_1.zip › Data Sheet 1/Supplementary Table S4.docx]

**Supplementary Table S4.** Distribution of distinct types of repeats in the 21 *Corydalis* cp genomes

| **S. No.** | **Species** | **Simple sequence repeat** | | | | | | | | | | | | | **Tandem repeat** | **Dispersed repeat** | | | | **Total** |
| --- | --- | --- | --- | --- | --- | --- | --- | --- | --- | --- | --- | --- | --- | --- | --- | --- | --- | --- | --- | --- |
|  |  | **A** | **C** | **G** | **T** | **AG** | **AT** | **CT** | **TA** | **ATG** | **ATT** | **CAA** | **TTA** | **TTG** |  | **F** | **R** | **C** | **P** |  |
| 1 | *C. adunca* | 20 | 1 | - | 15 | 1 | 2 | - | 1 | - | - | - | - | - | 71 | 27 | - | - | 23 | **161** |
| 2 | *C. conspersa* | 11 | 1 | - | 13 | - | 1 | - | - | - | - | - | - | - | 57 | 50 | - | - | - | **133** |
| 3 | *C. davidii* | 24 | 1 | - | 11 | - | 1 | - | 2 | - | - | - | - | - | 51 | 50 | - | - | - | **140** |
| 4 | *C. edulis* | 4 | 1 | - | 17 | - | - | - | 1 | - | - | 1 | - | - | 21 | 33 | 4 | 1 | 12 | **95** |
| 5 | *C. fangshanensis* | 9 | 1 | - | 20 | - | 3 | - | 6 | - | - | 1 | - | - | 40 | 49 | - | - | 1 | **130** |
| 6 | *C. filistipes* | 11 | 1 |  | 8 | - | 2 | - | 3 | - | - | - | 1 | - | 27 | 19 | - | - | 31 | **103** |
| 7 | *C. hsiaowutaishanensis* | 13 | 3 | 1 | 16 | - | 2 | - | 1 | - | - | - | - | - | 42 | 32 | - | - | 18 | **128** |
| 8 | *C. impatiens* | 15 | 1 | - | 17 | - | 4 | - | - | 1 | - | - | - | - | 55 | 49 | - | - | 1 | **143** |
| 9 | *C. inopinata* | 15 | 2 | - | 9 | - | 1 | - | - | - | - | - | - | - | 34 | 47 | - | - | 3 | **111** |
| 10 | *C. lupinoides* | 25 | 1 | 3 | 18 | - | 1 | - | - | - | 1 | - | - | - | 47 | 43 | 1 | - | 6 | **146** |
| 11 | *C. maculata* | 9 | 1 | - | 8 | - | 4 | - | - | - | - | - | - | - | 29 | 24 | - | - | 18 | **93** |
| 12 | *C. mucronifera* | 18 | - | - | 15 | - | 1 | 1 | 2 | - | - | - | - | - | 29 | 24 | - | - | 18 | **109** |
| 13 | *C. namdoensis* | 11 | 2 | - | 8 | - | 4 | - | - | - | - | - | - | - | 32 | 27 | - | - | 23 | **107** |
| 14 | *C. pauciovulata* | 22 | - | - | 25 | - | 2 | - | 2 | - | - | - | - | - | 59 | 43 | 5 | - | 2 | **106** |
| 15 | *C. platycarpa* | 16 | 1 | 1 | 17 | 1 | 4 | - | 3 | - | - | 1 | - | - | 50 | 41 | - | - | 9 | **144** |
| 16 | *C. saxicola* | 7 | 2 | - | 21 | - | 2 | - | 3 | - | - | - | - | - | 42 | 48 | - | - | 2 | **127** |
| 17 | *C. shensiana* | 12 | - | - | 12 | - | 1 | - | 1 | - | - | - | - | - | 18 | 35 | 4 | 1 | 14 | **98** |
| 18 | *C. ternata* | 8 | - | - | 8 | - | - | - | 2 | - | - | - | - | 1 | 41 | 46 | - | - | 4 | **110** |
| 19 | *C. tomentella* | 9 | 1 | - | 21 | - | 4 | - | - | - | - | - | - | - | 44 | 48 | - | - | 2 | **129** |
| 20 | *C. trisecta* | 17 | 1 | - | 15 | - | - | - | - | - | - | - | - | - | 66 | 36 | - | - | 14 | **149** |
| 21 | *C. turtschaninovii* | 4 | - | - | 9 | - | 1 | - | 5 | - | - | - | - | - | 28 | 17 | 13 | - | 20 | **97** |
