# Supplementary material for: Extensive reorganization of the chloroplast genome of Corydalis platycarpa: A comparative analysis of their organization and evolution with other Corydalis plastomes
Source: Front Plant Sci. 2022 Dec 9;13:1043740. doi: 10.3389/fpls.2022.1043740 (PMC10115153; doi:10.3389/fpls.2022.1043740)
Supplement: Supplementary Table 1 — List of taxa and GenBank accession numbers used in the phylogenetic and molecular clock analyses. [file DataSheet_1.zip › Data Sheet 1/Supplementary Table S1.docx]

**Supplementary Table S1**. List of taxa and GenBank accession numbers used in the phylogenetic and molecular clock analyses.

| S. N. | Species name | NCBI number |
| --- | --- | --- |
|  | *Corydalis adunca* | NC_057183 |
|  | *Corydalis conspersa* | NC_047208 |
|  | *Corydalis davidii* | NC_057184 |
|  | *Corydalis edulis* | NC_054239 |
|  | *Corydalis fangshanensis* | NC_058642 |
|  | *Corydalis filistipes* | MK264349 |
|  | *Corydalis hsiaowutaishanensis* | NC_057185 |
|  | *Corydalis impatiens* | NC_060862 |
|  | *Corydalis inopinata* | NC_052866 |
|  | *Corydalis lupinoides* | MZ157278 |
|  | *Corydalis maculata* | MK264348 |
|  | *Corydalis mucronifera* | MZ983400 |
|  | *Corydalis namdoensis* | MK264350 |
|  | *Corydalis pauciovulata* | MK264352 |
|  | *Corydalis platycarpa* | OP142703 (In this study) |
|  | *Corydalis saxicola* | NC_057186 |
|  | *Corydalis shensiana* | NC_054240 |
|  | *Corydalis ternata* | MK264347 |
|  | *Corydalis tomentella* | NC_060366 |
|  | *Corydalis trisecta* | NC_061916 |
|  | *Corydalis turtschaninovii* | MK264351 |
|  | *Lamprocapnos spectabilis* | NC_039756 |
|  | *Chelidonium majus* | NC_046829 |
|  | *Coreanomecon hylomeconoides* | NC_031446 |
|  | *Hylomecon japonica* | NC_045388 |
|  | *Macleaya microcarpa* | NC_039623 |
|  | *Meconopsis racemosa* | NC_039625 |
|  | *Papaver orientale* | NC_037832 |
|  | *Papaver rhoeas* | MF943221 |
|  | *Papaver somniferum* | NC_029434 |
|  | *Gymnospermium microrrhynchum* | NC_030061 |
|  | *Caulophyllum robustum* | NC_042221 |
|  | *Nandina domestica* | NC_008336 |
|  | *Berberis bealei* | NC_022457 |
|  | *Epimedium koreanum* | NC_029943 |
|  | *Diphylleia cymosa* | NC_037908 |
|  | *Jeffersonia diphylla* | NC_053385 |
|  | *Ranunculus cantoniensis* | NC_045920 |
|  | *Glaucidium palmatum* | NC_041539 |
|  | *Stephania japonica* | NC_029432 |
|  | *Akebia quinata* | NC_033913 |
|  | *Kingdonia uniflora* | NC_035873 |
|  | *Nicotiana tabacum* | NC_001879 |
