# Supplementary figures and images for: Extensive reorganization of the chloroplast genome of Corydalis platycarpa: A comparative analysis of their organization and evolution with other Corydalis plastomes
Source: Front Plant Sci. 2022 Dec 9;13:1043740. doi: 10.3389/fpls.2022.1043740 (PMC10115153; doi:10.3389/fpls.2022.1043740)

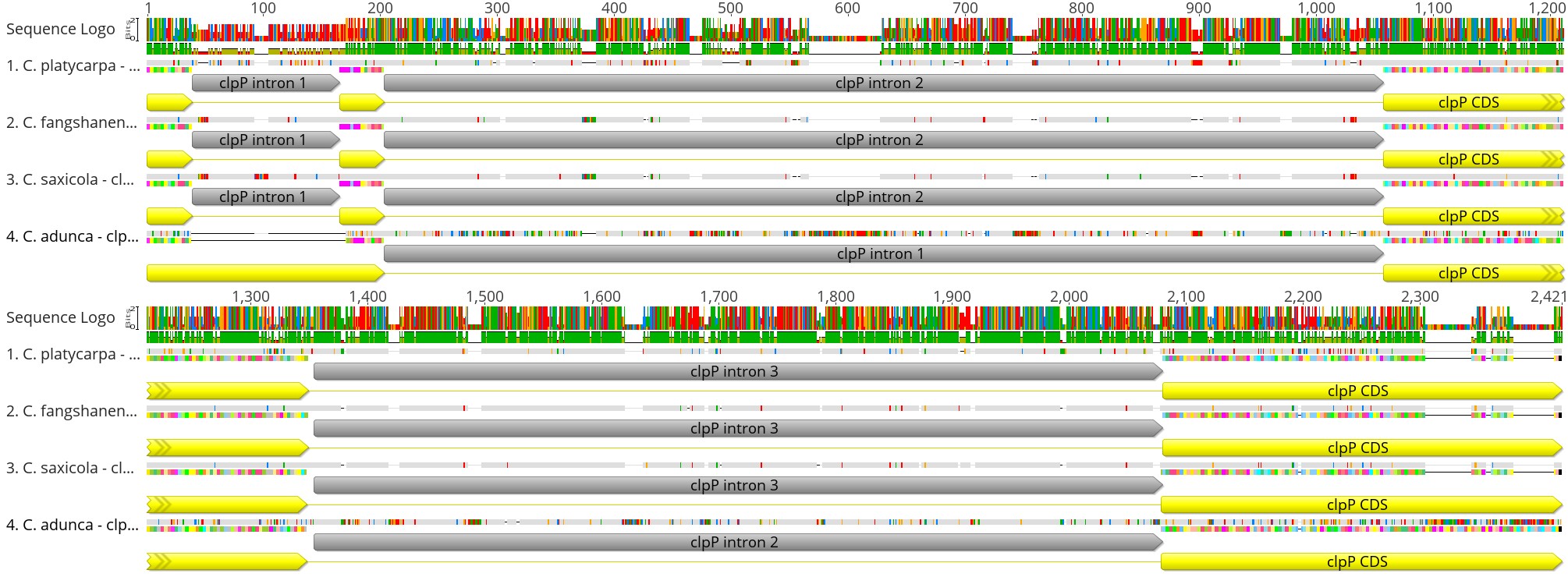

Supplement: Supplementary Table 1 — List of taxa and GenBank accession numbers used in the phylogenetic and molecular clock analyses. [file DataSheet_1.zip › Data Sheet 1/Supplementary Figure S4.jpg]

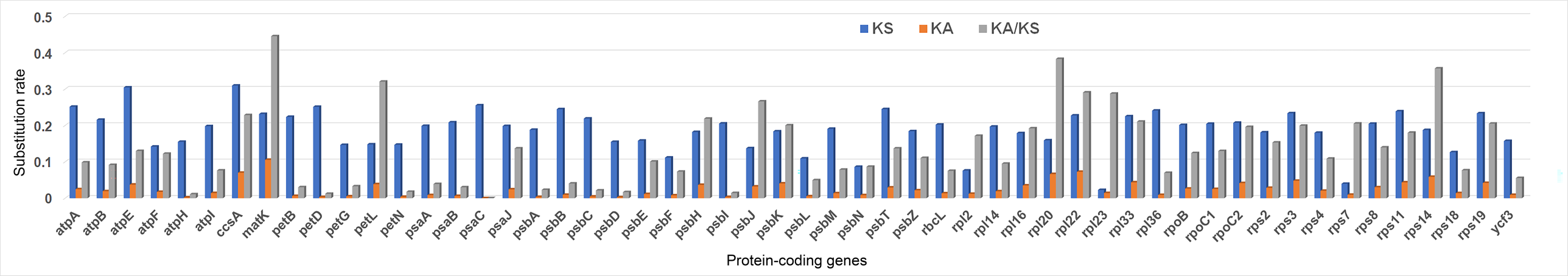

Supplement: Supplementary Table 1 — List of taxa and GenBank accession numbers used in the phylogenetic and molecular clock analyses. [file DataSheet_1.zip › Data Sheet 1/Supplementary Figure S3.jpg]

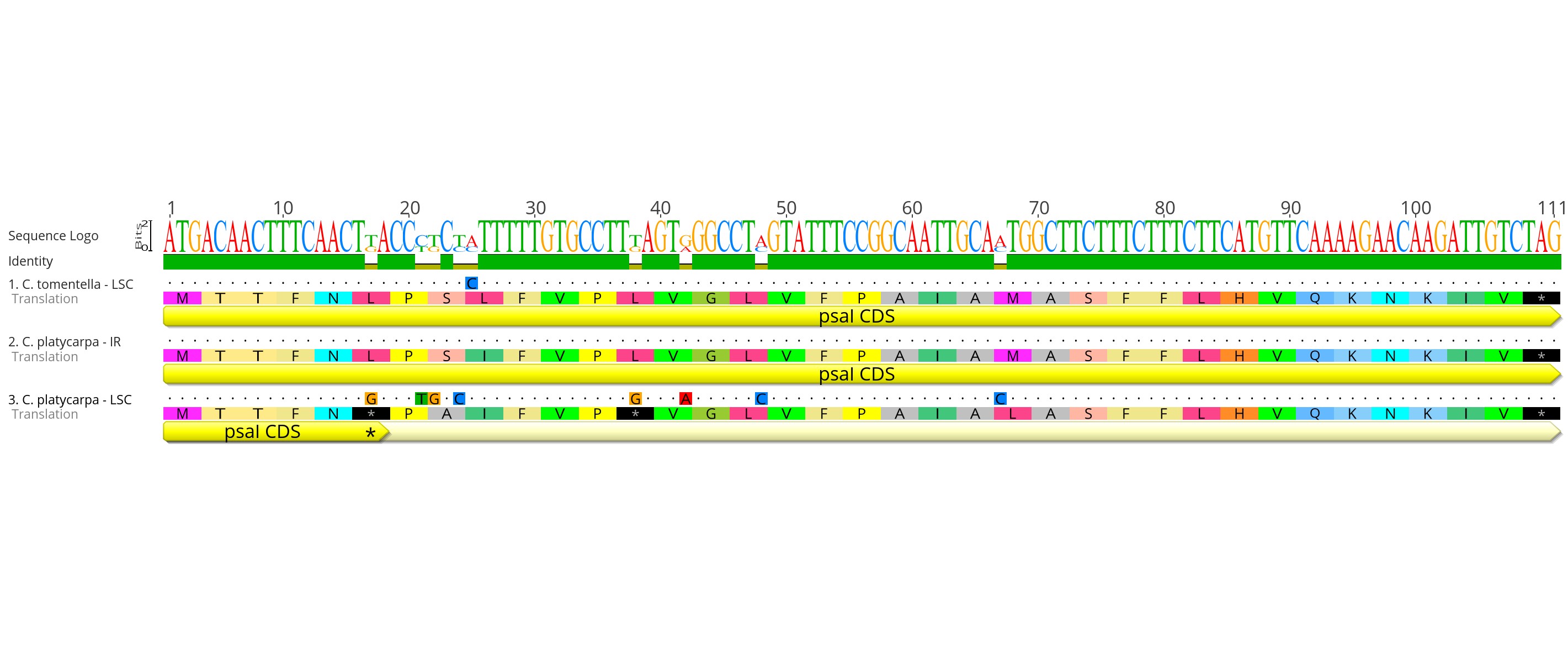

Supplement: Supplementary Table 1 — List of taxa and GenBank accession numbers used in the phylogenetic and molecular clock analyses. [file DataSheet_1.zip › Data Sheet 1/Supplementary Figure S5.jpg]

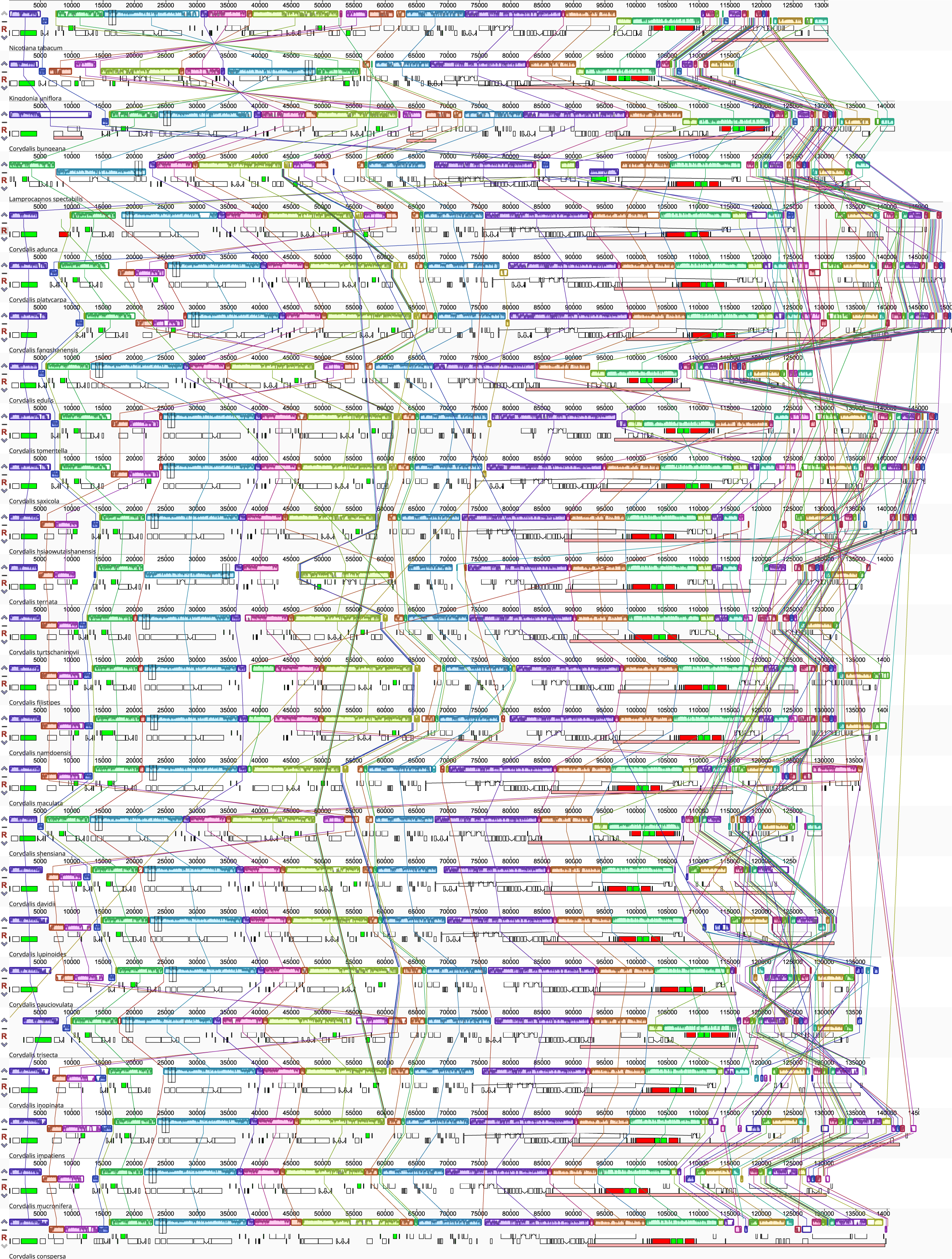

Supplement: Supplementary Table 1 — List of taxa and GenBank accession numbers used in the phylogenetic and molecular clock analyses. [file DataSheet_1.zip › Data Sheet 1/Supplementary Figure S1.tif]

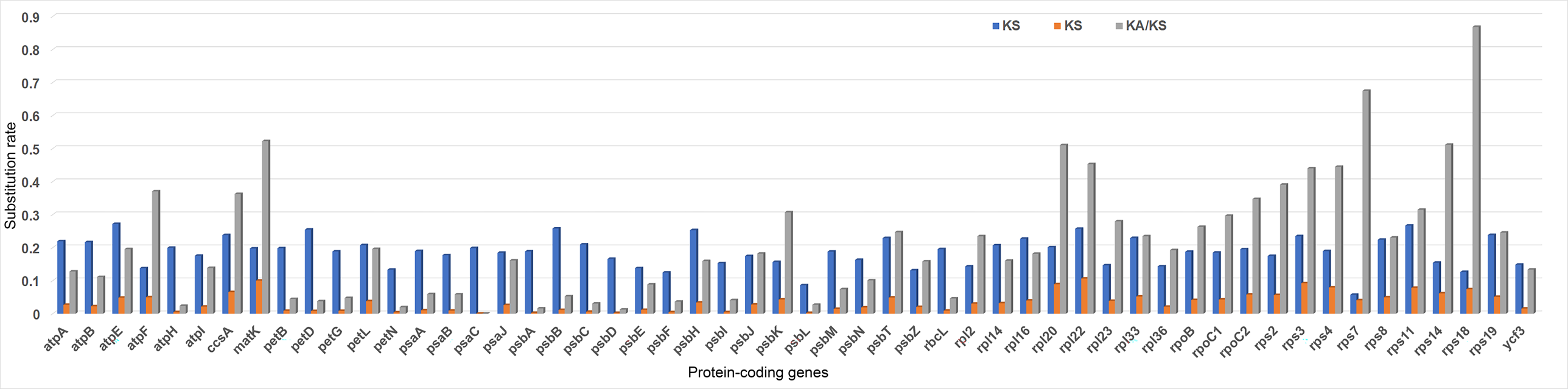

Supplement: Supplementary Table 1 — List of taxa and GenBank accession numbers used in the phylogenetic and molecular clock analyses. [file DataSheet_1.zip › Data Sheet 1/Supplementary Figure S2.jpg]
